# Polycomb dysregulation in gliomagenesis targets a *Zfp423*-dependent differentiation network

**DOI:** 10.1038/ncomms10753

**Published:** 2016-02-29

**Authors:** Elena Signaroldi, Pasquale Laise, Silvia Cristofanon, Arianna Brancaccio, Elisa Reisoli, Sina Atashpaz, Maria Rosa Terreni, Claudio Doglioni, Giancarlo Pruneri, Paolo Malatesta, Giuseppe Testa

**Affiliations:** 1 Department of Experimental Oncology, European Institute of Oncology, via Adamello 16, Milan 20139, Italy; 2 Trasferimento Genico, IRCCS-AOU San Martino-IST, Largo Rosanna Benzi 10, Genoa 16132, Italy; 3 Pathology Department, IRCCS San Raffaele Scientific Institute, via Olgettina 60, Milan 20132, Italy; 4 Division of Pathology and Laboratory Medicine, European Institute of Oncology, via Ripamonti 435, Milan 20141, Italy; 5 Department of Experimental Medicine (DiMES), University of Genoa, Via Leon Battista Alberti 2, Genoa 16132, Italy; 6 Department of Oncology and Hemato-oncology, University of Milan, Via Festa del Perdono 7, Milan 20122, Italy

## Abstract

Malignant gliomas constitute one of the most significant areas of unmet medical need, owing to the invariable failure of surgical eradication and their marked molecular heterogeneity. Accumulating evidence has revealed a critical contribution by the Polycomb axis of epigenetic repression. However, a coherent understanding of the regulatory networks affected by Polycomb during gliomagenesis is still lacking. Here we integrate transcriptomic and epigenomic analyses to define Polycomb-dependent networks that promote gliomagenesis, validating them both in two independent mouse models and in a large cohort of human samples. We find that Polycomb dysregulation in gliomagenesis affects transcriptional networks associated with invasiveness and de-differentiation. The dissection of these networks uncovers *Zfp423* as a critical Polycomb-dependent transcription factor whose silencing negatively impacts survival. The anti-gliomagenic activity of *Zfp423* requires interaction with the SMAD proteins within the BMP signalling pathway, pointing to a novel synergic circuit through which Polycomb inhibits BMP signalling.

## Abstract (web summary)

Polycomb-mediated gene regulation has been implicated in gliomas. Here the authors integrate transcriptomic and epigenomic analyses to define Polycomb-dependent networks that promote gliomagenesis, and find that the Polycomb-dependent silencing of the transcription factor *Zfp423* hinders survival.

## Graphical abstract

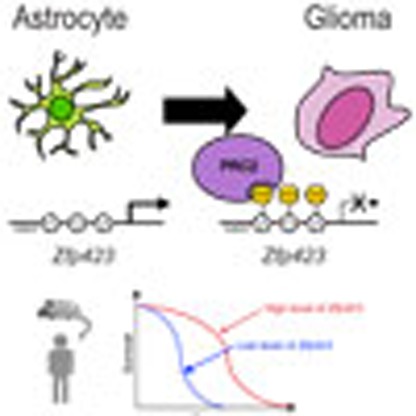

The Polycomb group (PcG) epigenetic axis highlights the intimate regulatory nexus between development and cancer, as its members play a critical role in the acquisition, maintenance and reversion of developmental fate^1^, whose alterations can in turn underlie the expansion or restoration of a dedifferentiated stem-like state that is thought to fuel the growth of many cancers^2^. Trimethylation of lysine 27 of histone H3 (H3K27me3), catalysed by EZH2 or EZH1 within Polycomb repressive complex 2 (PRC2), is both a major mechanism for the reversible maintenance of gene silencing^3^ and one of the few histone posttranslational modifications for which *bona fide* evidence supports a mechanism of propagation through cell division, thus providing the molecular basis for the maintenance of aberrant repressive chromatin during cancer growth^4^. In particular, convergent lines of evidence implicate Polycomb developmental functions as critical nodes of dysregulation during tumorigenesis. First, the majority of genes that are hypermethylated in cancers on CpG promoters are pre-marked by H3K27me3 during development, corroborating the model in which PcG-dependent programmes that orchestrate normal development can be hijacked in cancer as templates for aberrant DNA methylation^5^,^6^,^7^. Second, inappropriate expression of PcG proteins was directly shown to fuel dedifferentiation^8^, and more recently the dynamic acquisition of the bivalent chromatin configuration featuring both H3K27me3 and H3K4me3, which has been associated to both embryonic and tissue-specific stem cells^9^, was found to mediate the plastic interconversion, within the same tumour, of non-cancer stem cells into cancer stem cells^10^.

Furthermore, and beyond the developmental aspects of its function, the PcG system has been also implicated in other cardinal features of tumorigenesis, including silencing of the INK4A-ARF-INK4B locus^11^, promotion of angiogenesis^12^ and direct control of DNA replication^13^.

However, we still lack a coherent understanding of the genome-wide circuits affected by Polycomb during tumorigenesis, a deficit that has grown more relevant with accumulating evidence that PcG members can play opposing roles in tumorigenesis. Besides the ‘classical' oncogenic properties of EZH2 (refs 14, 15, 16), recent evidence has pointed also to a tumour suppressor role manifested through either inactivating mutations of PRC2 members^17^,^18^ or histone mutations that were shown to inhibit PRC2 activity^19^,^20^. Indeed, in the case of glioblastoma (GBM), although EZH2 is very frequently overexpressed, only its short-term inhibition ameliorates survival, while a longer depletion exacerbates tumour-related dedifferentiation^21^. Yet, although these results underscore the extreme context dependence of Polycomb involvement in tumorigenesis^22^, its molecular basis remains elusive. Even in settings in which EZH2 inhibition proved remarkably effective, as in lymphomas with gain-of-function mutations, it was not possible to define consistent transcriptional outputs, indicating that Polycomb can regulate different pathways even within the same apparently homogeneous tumour type^15^. This diversity of molecular impact is being linked to what is increasingly recognized, starting from Polycomb, as the core function of many chromatin modifiers, namely the definition of cell context-dependent ranges or thresholds of gene expression rather than the univocal determination of transcriptional output^2^,^23^. In turn, this grounds the rationale to pursue the elucidation of Polycomb-dependent programmes in highly defined and physiopathologically meaningful tumour models.

Here we address this challenge focusing on malignant gliomas, which constitute one of the most significant unmet medical needs and present two particularly relevant features: (i) pronounced heterogeneity among and within tumour types and (ii) convergent evidence of context-dependent Polycomb involvement, which is largely limited to its impact on single genes and has not mechanistically uncovered the diversity of its target pathways in relation to specific glioma subtypes and developmental contexts^24^,^25^,^26^,^27^,^28^,^29^,^30^,^31^. Specifically, we integrate transcriptomic and epigenomic analyses to define functionally relevant PRC2-dependent networks that promote gliomagenesis, validating them both in mouse models and human samples. We find that PRC2 affects specific developmental pathways associated to defining features of the disease. Moreover, the dissection of PRC2-dependent circuits at distinct stages of gliomagenic transformation uncovers *Zfp423* as a critical downstream target of PRC2, whose downregulation negatively impacts survival and whose overexpression impairs gliomagenesis in a SMAD (small mother against decapentaplegic)-dependent manner.

## Results

### Polycomb targets developmental pathways during gliomagenesis

**Figure 1:**
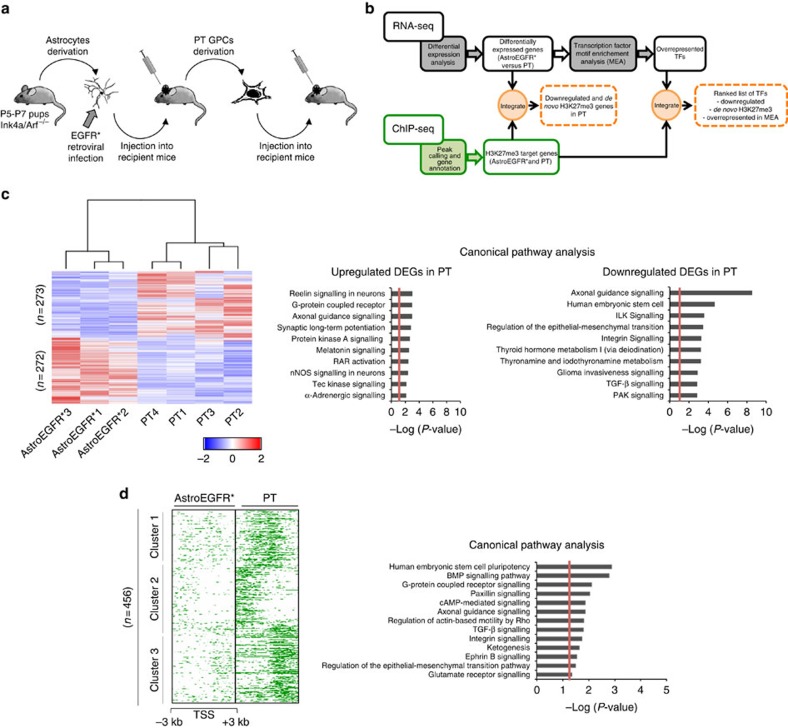
Transcriptional changes and H3K27me3 repositioning during gliomagenesis. (**a**) Schematic representation of the experimental procedure followed in this study. Briefly, astrocytes derived from cortices of *Ink4a/Arf*^−/−^ pups were infected with the human EGFR* and stereotaxically transplanted into recipient mice. GPCs were derived from PT and re-injected into secondary hosts to assess tumour propagation capability. (**b**) Schematic representation of the computational pipeline. RNAseq data were used to identify DEGs between *Ink4a/Arf*^−/−^ astrocytes overexpressing the human EGFR* (referred to as AstroEGFR*) and GPC (referred to as PT). ChIPseq data defined H3K27me3 target genes in the same cell populations. Integration of the results outlined a subset of genes that are significantly downregulated upon *de novo* H3K27me3 acquisition in PT. TF motif enrichment analysis (MEA) was performed on DEGs and integrated with RNAseq and ChIPseq data. (**c**) Left: heatmap of the DEGs between AstroEGFR* and PT. Colours represent *Z*-scores of the row-wise normalized expression values of log_2_ of fragments per kilobase of transcript per million mapped reads (FPKM; red: increased expression, blue: reduced expression). Right: enrichment analysis for canonical pathways in the DEGs. The probability (*P*-value) of random association between the gene set and given terms is calculated using the right-tailed Fisher's exact test. Only the top ten most significant pathways are reported; bar represents the −log (*P*-value). (**d**) Left: heatmap of normalized tag densities, representing the *de novo* methylated genes in PT. Each row represents a 6-kb window centred on the gene TSS and extending 3 kb upstream and 3 kb downstream. The signal has been generated by merging the bam files of the biological replicates. Right: enrichment analysis for canonical pathways in the same gene set.

To define the gene circuits that are deregulated during gliomagenesis in a PRC2-dependent manner, we used a well-established mouse model of gliomagenesis that relies on the loss of *Ink4a/Arf* combined with the overexpression of the constitutively active mutant form of the human epidermal growth factor receptor (EGFR) known as EGFR variant III (hereafter indicated as EGFR*), the two most common lesions in human high-grade gliomas (HGGs)^32^ (Fig. 1a and Supplementary Fig. 1a). The model relies on orthotopic transplantation of early postnatal astrocytes harbouring both lesions (hereafter *Ink4a/Arf*^*−/−*^; EGFR*) and its unique advantage, as depicted in Fig. 1a, is the definition of largely homogeneous cell populations endowed with different tumorigenic potential (non-tumorigenic primary *Ink4a/Arf*^*−/−*^ astrocytes, hereafter Astro; tumorigenic *Ink4a/Arf*^*−/−*^ astrocytes expressing EGFR*, hereafter AstroEGFR*; glioma propagating cells (GPCs) derived from primary tumours, hereafter PT; and GPCs derived from secondary tumours, hereafter ST), which constitute a privileged entry point to dissect the transcriptional and epigenomic alterations that accrue during gliomagenesis. Importantly, the *Ink4a/Arf*-null background allows to study the developmental features of PRC2-dependent oncogenesis independent of its control of the INK4A-ARF-INK4B locus^11^. We first validated the model through a thorough histopathological assessment, which extended previous observations^30^, confirming that in mouse the *Ink4a/Arf*^*−/−*^; EGFR* combination recapitulates a broad range of HGG, ranging from anaplastic astrocytomas, oligodendrogliomas and oligoastrocytomas to GBM and gliosarcoma (Supplementary Fig. 1b,c). Next, we focused on the key transition between AstroEGFR* and PT, and performed chromatin immunoprecipitation sequencing (ChIPseq) and RNA sequencing (RNAseq) for H3K27me3, to investigate the hypothesized redistribution of the PRC2 mark and its transcriptional impact during gliomagenesis. For this, we developed an *ad hoc* computational pipeline that integrates RNAseq and ChIPseq profiles with transcription factor (TF) motif enrichment analysis (Fig. 1b), to generate mechanistically testable hypotheses on the relevant Polycomb-dependent circuits.

A transcriptomic analysis of three independent *Ink4a/ARF*^*−/−*^;EGFR* astrocyte batches along with their resulting PT led to the identification of a set of 545 differentially expressed genes (DEGs), of which 272 are downregulated and 273 upregulated in PT versus AstroEGFR*. A cluster analysis based on DEGs revealed a clear clustering by cell type (that is, with tumorigenic astrocytes segregating away from all primary tumours; Fig. 1c). This pointed to a major and largely convergent shift in gene expression underlying the AstroEGFR*-PT transition. As shown in Fig. 1c (right), an Ingenuity Pathway Analysis (IPA) for canonical pathways uncovered the strongest enrichments among the downregulated DEGs, with a convergent involvement of genes controlling cell morphogenesis and migration, and belonging to the top scoring axonal guidance signalling, regulation of epithelial-to-mesenchymal transition (EMT) and integrin signalling pathways, whose key regulators have a well-established involvement in gliomas. Along with the related Reelin signalling pathway, axonal guidance signalling came out among the best-ranking pathways also within the upregulated DEGs (Fig. 1c), consistent with its dual role in regulating cell migration by fine-tuning both attractive and repulsive cues. Thus, molecules that are generally associated to repulsive clues, such as *Slit2–3* and *Shh*, appeared to be downregulated, whereas attractors such as *Wnt7b* and *Vegfa* were upregulated, consistently with the acquisition of the infiltrating capacity that characterizes malignant gliomas. Finally, the second top-scoring enriched pathway in downregulated DEGs was human embryonic stem cell (Fig. 1c), whose enrichment was due to the exclusive involvement of differentiation-promoting genes. This points to dedifferentiation as a particularly significant feature of gliomagenesis and further extends the insight that gliomas originate through the reversion of either astrocytes (as in our experimental system^32^) or neurons^33^ to an undifferentiated state. Consistently, we found that *Tgfb3*, known to promote neuronal differentiation^34^, *Bmp4*, involved in astrocyte differentiation^35^, and *Smad7*, important in neural fate acquisition^36^, were all downregulated in our PT samples.

When we analysed the same cell populations for H3K27me3 distribution, comparing the number of PRC2 targets in each class of samples revealed a major overall increase of PRC2 marking in PT, with 2,306 gene hits compared with 668 in AstroEGFR*. We then compared these gene sets with two previous data sets that captured distinct aspects of PRC2 developmental control: (i) the H3K27me3 signature that characterizes cortical neural progenitors (NPs)^37^, finding that although PT samples shared a statistically significant overlap of H3K27me3 target genes with NPs (92 genes), AstroEGFR* did not show any specific overlap (Supplementary Fig. 1e, left); and (ii) the core set of PRC2 targets whose silencing we found to be required for cell fate reversion to pluripotency^1^, finding that PT samples shared a striking overlap of H3K27me3 target genes with this core set (732 genes), whereas AstroEGFR* showed only a minimal overlap (12 genes) (Supplementary Fig. 1e, right). Together, these results highlight the extent of developmental dysregulation and dedifferentiation that characterize this tumour model. Next, to analyse the dynamics of PRC2 targets, we focused on the 456 genes that acquire the mark during the transition from AstroEGFR* to PT consistently across all PT samples (Fig. 1d, left). Also in this case, axonal guidance signalling and human embryonic stem cell pathways scored among the top ten in the IPA analysis of canonical pathway associated to this subset of genes (Fig. 1d, right). Importantly, *de novo* targets featured three clearly distinct enrichment patterns for H3K27me3: (i) that centred on the transcription start site (TSS), with the typical pattern of great enrichment right upstream and downstream of the sharp dip coinciding with the TSS^38^; (ii) that with a prevalent enrichment upstream of the TSS (−3 kb); or (iii) that with a prevalent enrichment downstream of the TSS (+3 kb). Comparison of expression values for genes belonging to each of the three classes showed a significant downregulation for those in which the accumulation of the mark included both the TSS and its downstream region (Supplementary Fig. 1f).

**Figure 2:**
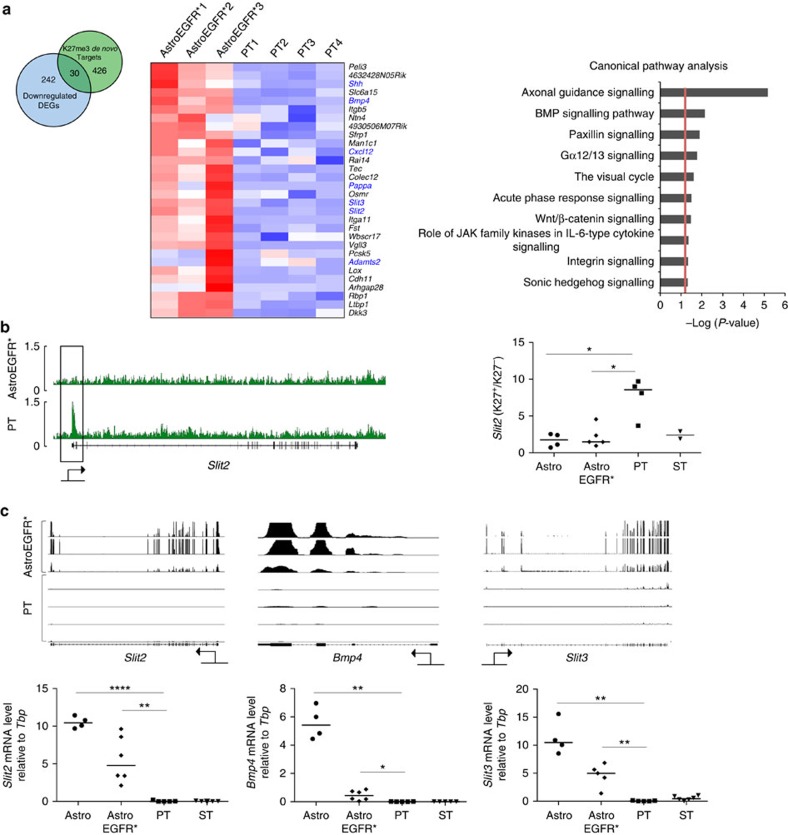
H3K27me3-mediated downregulation affects the axon guidance pathway. (**a**) Left: Venn diagram displaying the overlap between downregulated DEGs (light blue) and *de novo* H3K27me3 targets (light green) in PT. Middle: heatmap representing the gene expression changes between AstroEGFR* and PT of the 30 downregulated DEGs and *de novo* H3K27me3 targets. Genes belonging to the axon guidance pathway are written in blue. Right: enrichment analysis for canonical pathways in the same gene set. (**b**) Left: IGV visualization of the H3K27me3 ChIPseq tracks for the *Slit2* gene. Black rectangle highlights the peak on the TSS. Right: ChIP–qPCR validation of the H3K27me3 enrichment. Data represent the fold increase of the K27me3-positive region of the promoter normalized on the total H3 (K27^+^) over a K27me3-negative region in close proximity, again normalized on the total H3 (K27^−^). Each symbol represents a different sample for the indicated categories on the *x* axis (AstroEGFR*, *Ink4a/Arf*^−/−^ astrocytes overexpressing the human EGFR*; Astro, *Ink4a/Arf*^−/−^ astrocytes; PT, primary tumour GPC; ST, secondary tumour GPC); black bars within the symbols represent the median (*P<0.05) (*P*-values were calculated using the Mann-Whitney test). (**c**) Top: IGV visualization of the RNAseq tracks for selected genes of the axon guidance pathway. Bottom, analysis of transcript levels measured by qRT–PCR for the same genes. Data are represented as dCt (log_2_ scale) relative to *Tbp*. Each symbol represents a different sample in the specified categories on the *x* axis; black bars within the symbols represent the median. (*P<0.05, **P<0.01 and ****P<0.0001) (*P*-values were calculated using the Mann–Whitney test).

Finally, we integrated the RNAseq and ChIPseq data sets to pinpoint the genes that, in the AstroEGFR*–PT transition, became both downregulated and *de novo* targets of H3K27me3 (Fig. 2a, left). Remarkably, axonal guidance signalling was strongly associated with this subset of genes as well, indicating that this pathway is under direct Polycomb control during the gliomagenic transition (Fig. 2a, right). Figure 2b,c shows the independent validation of these findings for representative members of the axonal guidance signalling pathway, namely *Slit2*, *Slit3* and *Bmp4*, which were tested for H3K27me3 enrichment (Fig. 2b) and expression (Fig. 2c). *Slit2* and *Slit3* are two key molecules originally involved in axon guidance (in which they provide repulsive cues by binding to their Roundabout receptors)^39^,^40^, whose functional association with glioma emerged with the observation that *Slit2* is epigenetically silenced in gliomas through DNA methylation^41^, and that rescue of its expression reduces cell migration both *in vitro* and *in vivo*^42^. *Bmp4* is known to reduce proliferation and promote astrogenesis^35^; hence, its silencing further highlights dedifferentiation as a core feature of gliomagenesis. Interestingly, the core subset of genes that are both downregulated and acquire *de novo* H3K27me3 in the AstroEGFR* to PT transition showed a highly significant overlap with the subset of Polycomb targets that are significantly downregulated during cell reprogramming (Supplementary Fig. 2a). Among these genes, we found *Bmp4* along with other genes involved in cellular differentiation, further emphasizing the key role played by Polycomb during the transcriptional shift from astrocytes to primary tumours (Supplementary Fig. 2b).

### *Zfp423* is a master Polycomb target during gliomagenesis

**Figure 3:**
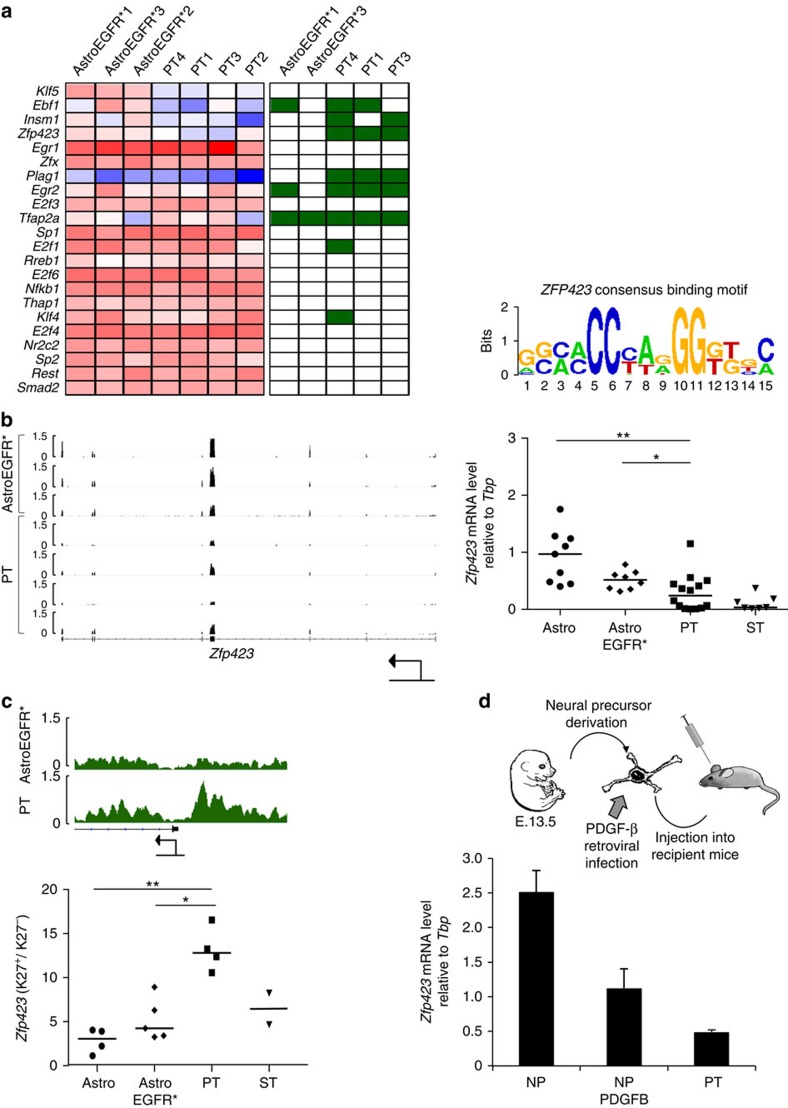
*Zfp423* is downregulated in PT upon H3K27 trimethylation and its binding motif is overrepresented among DEG promoters. (**a**) Left: heatmap representing the gene expression levels (red: increased expression, blue: reduced expression) between AstroEGFR* and PT of the most significant, overrepresented TFs identified with *P*scan (Bonferroni *P*-value<0.05). The second heatmap represents H3K27me3 status of the promoter of the same genes (white: unmethylated, green: methylated). Right: *Zfp423*-binding motif. (**b**) Left: IGV visualization of the RNAseq tracks for *Zfp423*. Right: analysis of transcript levels measured by qRT–PCR in different cell populations. Data are represented as dCt (log_2_ scale) relative to *Tbp*. Each symbol represents a different sample for the specified category; black bars within the symbols represent the median. (*P<0.05 and **P<0.01) (*P*-values were calculated using the Mann–Whitney test). (**c**) Top: IGV visualization of the H3K27me3 ChIPseq tracks for the *Zfp423* gene. Bottom: ChIP–qPCR validation of the H3K27me3 enrichment. Data represent the fold increase of the K27me3-positive region of the promoter normalized on the total H3 (K27^+^) over a K27me3-negative region in close proximity, again normalized on the total H3 (K27^−^). Each symbol represents a different sample in each category; black bars within the symbols represent the median (*P<0.05 and **P<0.01) (*P*-values were calculated using the Mann–Whitney test). (**d**) Top: schematic representation of a different glioma mouse model in which *Zfp423* downregulation was confirmed. Neural precursors (NPs) were isolated from telencefalic vesicles of embryos at 13.5 dpc. Cells were infected with the mouse *Pdgf-β* and transplanted stereotaxically into recipient mice. GPCs were derived from PTs. Bottom: *Zfp423* messenger RNA levels were measured in the different cell populations by qRT–PCR. Data are represented as dCt (log_2_ scale) relative to *Tbp*; error bars represent the s.d. of the technical replicates.

PRC2 had thus far been implicated in gliomagenesis on the basis of either clinical correlations^29^ or of its effect on exemplary genes involved in astroglial differentiation^28^. By defining a consistent genome-wide pattern of PRC2 redistribution across independent glioma samples and characterizing its transcriptional output, our results enabled a first dissection of the specific contribution of this epigenetic axis, uncovering two prominent domains of developmental dysregulation (that is, the control of migration and differentiation outlined above). However, beyond these specific pathways and consistent with recent evidence^2^, this approach also confirmed that only a minor portion of the extensive transcriptional changes that accrue during gliomagnesis could be directly imputable to aberrant PRC2 occupancy. Thus, to gain further mechanistic insight into the functional relevance of H3K27me3 redistribution, we focused on the identification of putative PRC2-dependent master regulator TFs that could mainly and indirectly account for the transcriptional changes we observed. For this, we first performed a TF motif enrichment analysis of the DEGs between AstroEGFR* and PT. This analysis pinpointed 22 candidate master regulator TFs, defined as TFs whose binding sites were significantly enriched among the DEGs characterizing the AstroEGFR* to PT transition. These genes were then ranked according to the significance of their expression change and correlated to their H3K27me3 status. As shown in Fig. 3a, among the 22 predicted candidate master regulator TFs, *Zfp423* was the only one whose repression correlated with the *de novo* acquisition of H3K27me3 in PT. Furthermore, meta-analysis of an RNAseq data set obtained from a different murine glioma model^21^ revealed an inverse expression correlation between the PRC2 enzymatic subunit *Ezh2* and *Zfp423*, with *Ezh2* knockdown resulting in increased *Zfp423* expression (Supplementary Fig. 3a). We next confirmed both the downregulation (Fig. 3b) and the *de novo* H3K27me3 acquisition of *Zfp423* (Fig. 3c) in a larger cohort of samples, highlighting the consistency of this silencing circuit.

As recounted above, gliomas display remarkable heterogeneity both at the histopathological and molecular level. In addition, we thus analysed a different mouse model that is based on the overexpression of platelet-derived growth factor beta (PDGF-B) in cortical NP (see ref. 43 and Fig. 3d), to determine whether the glioma-associated repression of *Zfp423* was consistent across different tumour-driving mutations. In this case as well, we characterized homogeneous populations of cells that captured different disease stages, namely primary, non-tumorigenic NP sourced from E13.5 telencephali (hereafter NP), tumorigenic NP overexpressing PDGF-B (hereafter NP PDGFB) and GPCs derived from primary tumours (hereafter PT) that were able to regrow tumours upon orthotopic transplantation. Consistent with our observations from the *Ink4a/Arf*^*−/−*^; EGFR* model, we confirmed a progressive downregulation of *Zfp423* along with the increase in tumorigenic potential (Fig. 3d).

### *Zfp423* is downregulated in human gliomagenesis

**Figure 4:**
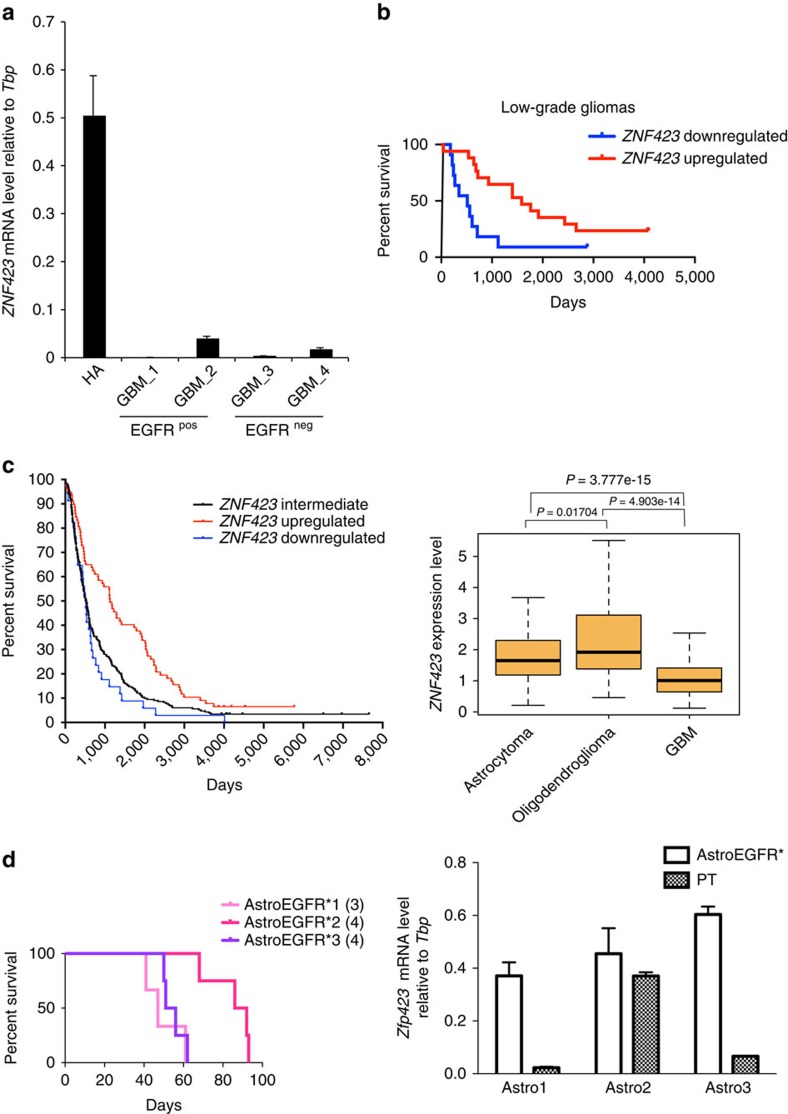
Strong downregulation of *Zfp423* correlates with poor prognosis. (**a**) mRNA levels of the human orthologue of *Zfp423*, *ZNF423*, were measured in human glioma samples classified according to EGFR expression (GBM, GBM sample; HA, human astrocytes) by qRT–PCR. Data are represented as dCt (log_2_ scale) relative to *TBP*; error bars represent the s.d. of technical replicates. (**b**) Kaplan–Meyer analysis of survival in low-grade glioma patients with high (red, *n*=17) or low (blue, *n*=11) *ZNF423* expression levels (*P*<0.01, data obtained from The Cancer Genome Atlas, TCGA). (**c**) Survival and expression analysis of a large data set from the REpository for Molecular BRAin Neoplasia DaTa (REMBRANDT). Left: Kaplan–Meyer analysis of survival in patients with high (red, *n*=77), intermediate (black, *n*=232) or low (blue, *n*=34) *ZNF423* expression levels. Difference in progression-free survival between *ZNF423* high- and low-expressing patients is statistically significant (*P*=1.65218e−5). Right: boxplots represent expression levels of *ZNF423* in different samples grouped according to histopatological classification (*P*-values were computed with Mann–Whitney test). (**d**) Left: Kaplan–Meyer analysis of survival in mice injected with three representative AstroEGFR* cell lines (*n*=3 for AstroEGFR*1, *n*=4 for AstroEGFR*2 and *n*=3 for AstroEGFR*3). Right: *Zfp423* mRNA levels in the same three AstroEGFR* cell lines (white bars) and in primary GPC derived from corresponding tumours (black bars). Data are represented as dCt (log_2_ scale) relative to *Tbp*; error bars represent the s.d. of technical replicates.

Although *Zfp423* had not yet been associated to gliomas, a previous study^44^ found the human orthologue of *Zfp423*, *ZNF423*, to be downregulated in human neuroblastoma. We thus assessed the expression of *ZNF423* in well-defined human GBM samples that had been previously subdivided according to EGFR expression^45^ and found it consistently downregulated, regardless of EGFR levels, with respect to normal human astrocytes (Fig. 4a), thus confirming the observation from both mouse models. We then probed a large set of clinical data available from TCGA database (http://cancergenome.nih.gov/), to investigate the correlation between *ZNF423* expression and patient survival. To this end, we interrogated separately low-grade gliomas and GBMs, and stratified samples according to the expression of *ZNF423*. This analysis revealed a significant direct correlation between *ZNF423* expression and survival in low-grade gliomas (Fig. 4b), with high expression associated to longer survival. The correlation held also in GBM samples, although in this more malignant subset the trend fell short of the statistical significance threshold (Supplementary Fig. 4a).

In the case of neuroblastoma, *ZNF423* displayed a clear trend of progressively decreased expression with more advanced tumour stages and lower expression was associated with worst prognosis. Thus, to generalize our findings and test whether *ZNF423* behaved similarly in gliomas, we performed a meta-analysis of a large cohort of expression data from 343 samples of human gliomas deposited at the NCI REpository for Molecular BRAin Neoplasia DaTa^46^. As shown in Fig. 4c, we observed that also in gliomas, *ZNF423* downregulation correlated with higher grade (right panel). Comparable results were obtained by performing a second meta-analysis on a further independent cohort of expression profiles, comprising 157 expression data from Gene Expression Omnibus (GEO ID: GSE4290; Supplementary Fig. 4b). Moreover, when we classified patients from the REpository for Molecular BRAin Neoplasia DaTa database into three groups according to *ZNF423* expression on the basis of their fold change with respect to median expression, a Kaplan–Meyer analysis for progression-free survival confirmed that higher levels of *ZNF423* correlate with a far better prognosis compared with mid or low levels (Fig. 4c, left panel), with high statistical significance between high- (f.c.>2) and low-expressing (f.c.<2) tumours (Mantel–Haenszel test, *P*=1.65218e−5). Finally, to ground the mechanistic dissection of the role of *Zfp423* dosage in gliomagenesis, we resorted back to our initial *Ink4a/Arf*^*−/−*^; EGFR* murine model and interrogated three independent batches of tumorigenic astrocytes (AstroEGFR*1, AstroEGFR*2 and AstroEGFR*3) with comparable *Zfp423* expression. When challenged in *in-vivo* experiments upon orthotopic transplantation, we found that AstroEGFR*1 and AstroEGFR*3 yielded gliomas with a shorter survival compared with AstroEGFR*2 (Fig. 4d). Strikingly, although primary tumours originated from every astrocyte batch invariably displayed *Zfp423* downregulation, they did so to different extents, with AstroEGFR*1 and AstroEGFR*3 showing a greater reduction than AstroEGFR*2 (Fig. 4d), thus confirming results from human data and validating this model as a sensitive system to test the physiopathologically relevant role of *Zfp423*.

### Context-specific antigliomagenic effect of *Zfp423*

**Figure 5:**
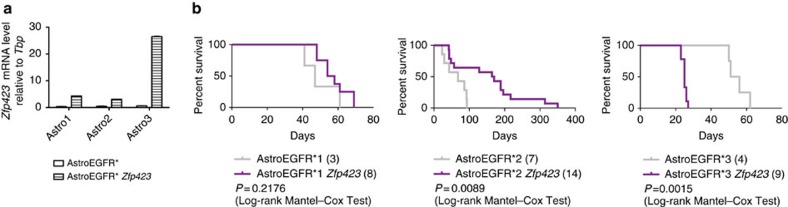
*Zfp423* overexpression differentially impacts survival. (**a**) AstroEGFR* cell lines were infected with retroviral vectors expressing *Zfp423* and *Zfp423* overexpression levels were then measured by qRT–PCR. Data are represented as dCt (log_2_ scale) relative to *Tbp*; error bars represent the s.d. of technical replicates. (**b**) Kaplan–Meyer analysis of survival in mice injected with the three batches of AstroEGFR* (light grey) and AstroEGFR* overexpressing *Zfp423* (purple). Numbers of animals for each category are indicated in brackets in the caption. Difference in progression-free survival between animals injected with AstroEGFR*2 and AstroEGFR*2 *Zfp423* is statistically significant.

As higher levels of *Zfp423* were associated to better prognosis in both humans and mice, we set out to validate its role by forcing its overexpression in tumorigenic *Ink4a/Arf*^*−/−*^; EGFR* astrocytes and thereby test whether preventing its PRC2-mediated downregulation would impair or delay gliomagenesis. The three representative independent batches of tumorigenic astrocytes, AstroEGFR*1, AstroEGFR*2 and AstroEGFR*3, were thus infected with a retroviral vector encoding *Zfp423* (ref. 47; hereafter AstroEGFR* *Zfp423*). Following confirmation of overexpression (Fig. 5a), cells were then assessed for tumorigenicity by sterotactic inoculation. Interestingly, the three AstroEGFR* lines responded differently to the challenge of *Zfp423* overexpression (Fig. 5b). As shown in Fig. 5b, the overexpression of *Zfp423* had a major impact on the tumorigenicity of AstroEGFR*2 astrocytes, with a highly statistically significant increase in survival (*P*=0.0089). On the contrary, mice transplanted with either AstroEGFR*1 or AstroEGFR*3 astrocytes showed different survivals. Specifically, although AstroEGFR*1, which reached overexpression levels comparable to those in AstroEGFR*2, showed a trend of extended survival that did not reach statistical significance, AstroEGFR*3, despite much stronger *Zfp423* overexpression, displayed a shorter survival. To gain insight into the molecular underpinnings of the differential response to *Zfp423* overexpression, we followed two complementary approaches.

**Figure 6:**
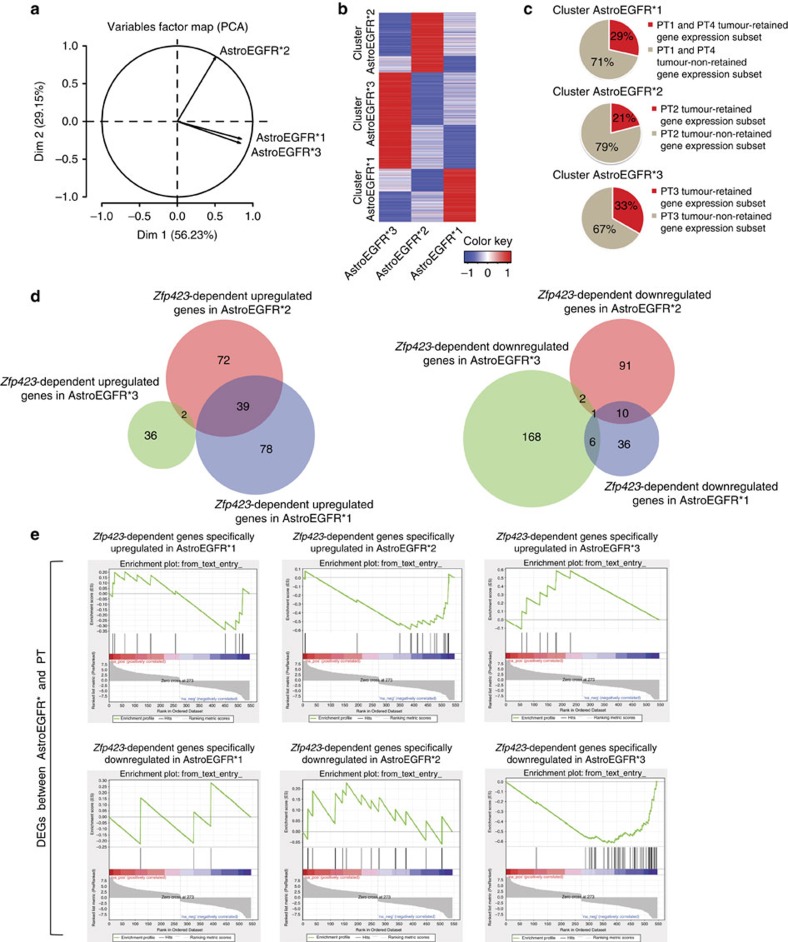
*Zfp423* overexpression differentially affects AstroEGFR* networks. (**a**) Variable factor map (principal component analysis (PCA)) representing the degree of correlation between AstroEGFR* transcriptomes. The PCA shows that AstroEGFR* cluster according to their responsiveness to *Zfp423* overexpression. Correlation coefficients of AstroEGFR*1, AsrtoEGFR*2 and AstroEGFR*3 with the first component are 0.8499869, 0.5120449 and 0.8380024, respectively. (**b**) Heatmap representing the clusters of genes selectively enriched in the three batches of tumorigenic astrocytes. (**c**) Pie charts representing the proportion of tumour-retained gene expression for each AstroEGFR*-derived PT. (**d**) Venn diagrams showing the overlap of *Zfp423*-dependent genes identified in the three different astrocytes batches upon *Zfp423* overexpression. (**e**) Gene set enrichment analysis showing the enrichment, within the DEGs initially identified between AstroEGFR* and PT, for *Zfp423*-dependent genes. The interrogation with the *Zfp423*-dependent genes upregulated in AstroEGFR*2 shows a significant enrichment in the DEGs that are downregulated in the gliomagenic transition from tumorigenic astrocytes to primary gliomas. Black bars represent the position of *Zfp423*-dpendent genes in the ranked list of DEGs between AstroEGFR* and PT. The green line represents the enrichment score. The nominal *P*-values associated with the *Zfp423*-dependent genes upregulated in AstroEGFR*2, the *Zfp423*-dependent genes upregulated in AstroEGFR*3 and the *Zfp423*-dependent genes downregulated in AstroEGFR*3 are statically significant (*P*<0.01).

First, we performed a principal component analysis of the AstroEGFR* transcriptomes, which revealed that AstroEGFR* cluster according to their responsiveness to *Zfp423* overexpression (Fig. 6a). Differential expression analysis uncovered gene clusters that were distinctly associated to each batch (Fig. 6b), which we then used to query PT transcriptomes finding that PT originated from AstroEGFR*1 and AstroEGFR*3, when compared with the AstroEGFR*2-derived PT, retained expression of a larger portion of the gene cluster belonging to the respective astrocyte batch (Fig. 6c). On the contrary, a shuffling analysis that randomly queried astrocyte batch-specific clusters against PT did not reveal the same degree of retention (Supplementary Fig. 5a). This establishes that a significant fraction of the gene expression that distinguishes similar yet distinct batches of tumorigenic astrocytes is retained in the respective tumours. Furthermore, it highlights how such tumour-retained gene expression is already primed to a greater extent in the *Zfp423-*resistant astrocyte batches that display, accordingly, a more aggressive tumorigenicity. This finding was strengthened by an unsupervised cluster analysis of the transcriptomes of the three AstroEGFR* and the respective PT. Indeed, each AstroEGFR* clusters with the corresponding tumour (Supplementary Fig. 5b), indicating that each AsroEGFR* batch is already seeded with partially specific transcriptional programmes that will unfold during tumour development.

Second, we compared by RNAseq the transcriptomes of the three batches of AstroEGFR* to their respective transcriptomes upon forced *Zfp423* expression (AstroEGFR*1 versus AstroEGFR*1 *Zfp423*, AstroEGFR*2 versus AstroEGFR*2 *Zfp423* and AstroEGFR*3 versus AstroEGFR*3 *Zfp423*) and went on to define the *Zfp423-*centred transcriptional interactions specific to each pair. To this end, we integrated the results of differential expression obtained from each comparison with a co-expression-based analysis extracted from a large data set of 106 gene expression profiles collected in the GeneMania database^48^. The aim was first to infer the high confidence co-expression networks centred on *Zfp423* and then define which portion of these predicted circuits were empirically validated in *Zfp423*-responsive versus *Zfp423*-non-responsive tumorigenic astrocytes. Consistent with the *in-vivo* outcome, interrogation of these gene networks showed that *Zfp423* overexpression had affected different sets of genes in the three batches of tumorigenic astrocytes (Fig. 6d). We thus reasoned that these distinct gene subsets should display marked differences with respect to the transcriptional programme that underlies primary gliomagenesis in the same murine model. To test this hypothesis, we thus compared, by gene set enrichment analysis, the *Zfp423*-dependent genes specifically affected in each batch of tumorigenic astrocyte with the DEGs that marked the AstroEGFR*-to-PT transition. This approach confirmed that the genes downregulated in the AstroEGFR*-to-PT transition were significantly enriched for the *Zfp423*-dependent genes that were upregulated specifically in AstroEGFR*2, providing a clear molecular basis for the antigliomagenic effect of *Zfp423* in this context. On the contrary, although we did not find any significant enrichment for *Zfp423*-dependent genes affected in AstroEGFR*1, we found a significant enrichment for the *Zfp423*-dependent genes affected in AstroEGFR*3, but among the genes that changed in the same direction as the DEGs characterizing the AstroEGFR*-to-PT transition (Fig. 6e), again providing the molecular correlate for the lack of antigliomagenic effect by *Zfp423* in this context. Together, these results provide a molecular basis for the antigliomagenic impact of *Zfp423* in the specific cellular context of AstroEGFR*2, grounding it in the ability to specifically antagonize a significant portion of the gliomagenesis-specific transcriptional programme that characterizes this murine model.

**Figure 7:**
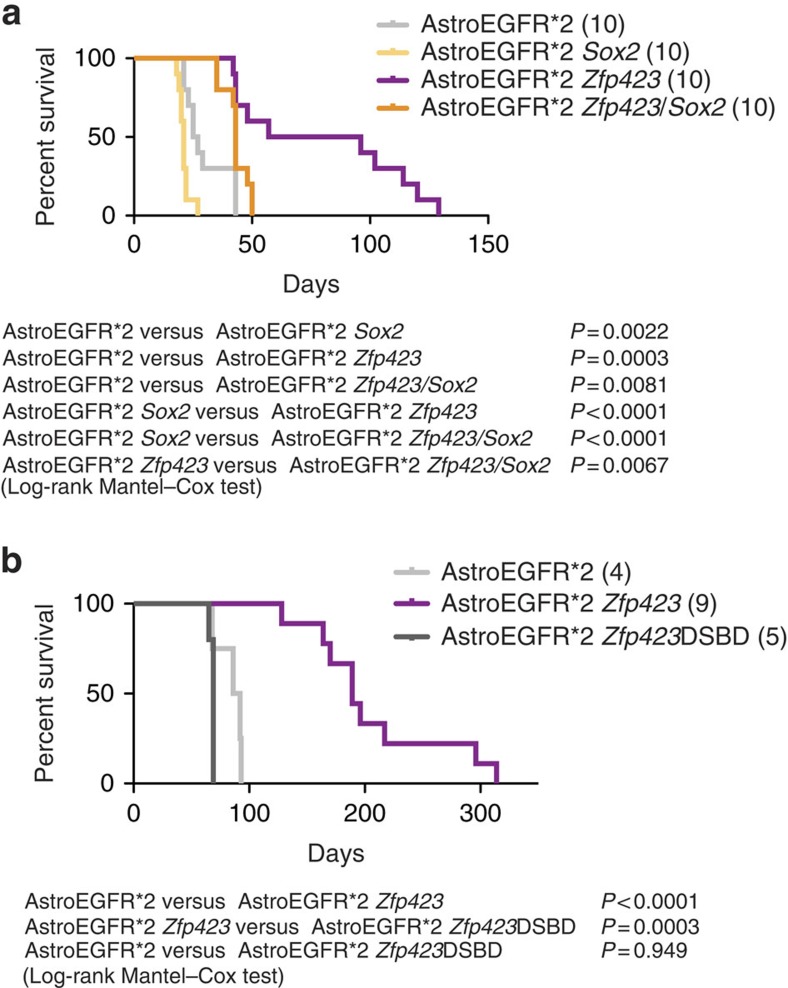
*Zfp423* expression antagonizes gliomagenesis in a SMAD-dependent manner. (**a**) Kaplan–Meyer analysis of survival in mice injected with AstroEGFR*2 overexpressing either *Zfp423*, *Sox2* or both. Reconstitution of *Sox2* expression in the context of *Zfp423* overexpression reverted the *Zfp423-*dependent antigliomagenic phenotype (orange line), leading to shorter survival compared with *Zfp423* overexpression alone (purple line). Overexpression of both *Zfp423* and *Sox2* also prolonged survival with respect to *Sox2* overexpression alone, indicating mutual antagonism between the two factors. Numbers of animals for each category are indicated in brackets in the caption; *P*-values were computed with log-rank Mantel–Cox test. (**b**) Overexpression of a mutant form of *Zfp423* lacking the SMAD-binding domain (*Zfp423Δ*SBD, dark grey curve) fails to prolong survival in mice. Numbers of animals for each category are indicated in brackets in the caption; *P*-values were computed with log-rank Mantel–Cox test.

Interestingly, among the genes specific to the *Zfp423* response there are several members of the axon guidance pathways and tumour suppressor genes whose modulation was already associated to reduced tumorigenicity: namely *Slit2* (refs 41, 42), *Pappa*^49^, *Nfatc4* (ref. 50) and *Lox*^51^. Furthermore, the group of genes specifically downregulated upon *Zfp423* overexpression only in AstroEGFR*2 and not in AstroEGFR*1 or AstroEGFR*3 included also *Sox2* and several other Sox family genes (Supplementary Fig. 5c), which have recently emerged as important driving or enabling factors in gliomagenesis^52^,^53^. As TF motif analysis predicted that the *Sox2* promoter contains a putative binding site for *Zfp423*, we asked whether *Zfp423* overexpression could antagonize gliomagenesis by repressing *Sox2* in *Zfp423*-responsive astrocytes. To test this hypothesis, we overexpressed *Sox2* in *Zfp423*-responsive astrocytes to revert the phenotype of extended survival, using a construct that lacked endogenous control regions and thus could not be subjected to *Zfp423* transcriptional repression. Indeed, the overexpression of *Sox2* in the context of *Zfp423* overexpression showed a survival rate that was much shorter compared with that achieved with *Zfp423* overexpression alone (Fig. 7a), thus establishing *Sox2* downregulation as a fundamental antigliomagenic event downstream of *Zfp423* overexpression. Interestingly, however, against the expected accelerated tumorigenesis induced by overexpression of *Sox2* alone, mice receiving astrocytes overexpressing both TFs showed longer survival (Fig. 7a), uncovering also a mutually antagonistic indirect circuit between *Zfp423* and *Sox2* in gliomagenesis.

Finally, in light of these analyses indicating that the *Ink4a/Arf*^*−/−*^; EGFR* paradigm is more diverse than previously anticipated, we investigated this diversity considering the pronounced heterogeneity characterizing human HGG, as captured in recent molecular classifications^24^,^25^,^26^. Specifically, we used the signatures defined by Phillips *et al.*^24^ to interrogate our cohort of PT samples and determine the extent to which they fit this classification, while measuring the spread of their molecular diversity. Notably, most murine models of glioma have not yet been interrogated with human signatures; thus, this analysis also provided a first characterization of the human equivalence, at the molecular level, of the *Ink4a/Arf*^*−/−*^; EGFR* model. We found that although no PT fit neatly into either of the three categories (proneural, proliferative or mesenchymal), each encompassed different partitions of the three signatures, with a prevalence of the mesenchymal one (Supplementary Fig. 5d). Interestingly, most of these partitions showed opposite trends in different samples, pointing to a fundamental level of heterogeneity that accompanies, in a preferentially mesenchymal background, *Ink4a/Arf*^*−/−*^; EGFR*-driven gliomagenesis.

### The *Zfp423* antigliomagenic role engages BMP-SMAD signalling

*Zfp423* is widely involved in differentiation^47^,^54^,^55^,^56^,^57^,^58^,^59^,^60^. Consistently, we had previously found it greatly downregulated upon reprogramming of mouse embryonic fibroblasts into induced pluripotent stem cells^1^. Of special relevance, *Zfp423* belongs also to the genes specifically expressed in the astrocyte lineage^61^, a finding that we further corroborated by inducing astrocytic differentiation and confirming the expected BMP4-enhanced upregulation of *Zfp423* (Supplementary Fig. 6a).

ZFP423 exerts its differentiation-promoting function by binding, through zinc fingers 14–20, to SMAD proteins^62^,^63^ that mediate response to bone morphogenetic protein (BMP) signalling in different cell types^62^ (Supplementary Fig. 6b). In our PT samples, both *Bmp4* and *Zfp423* are *de novo* H3K27 trimethylated and downregulated, pointing to a double and synergic blockade of the BMP signalling pathway that is critical for astroglial differentiation. Thus, also in light of the observation that *Zfp423* can coordinate BMP and Notch signalling throughout neural development, and that this effect is enhanced after BMP4 stimulation^63^, we infected the *Zfp423*-sensitive astrocytes (that is, AstroEGFR*2) with a well-characterized mutant isoform of *Zfp423* lacking the SMAD-binding domain^47^ (SBD, hereafter *Zfp423*ΔSBD) and assessed their tumorigenicity *in vivo*. Contrary to the major antigliomagenic effect observed with the overexpression of the full-length protein, overexpression of mutant *Zfp423* did not affect the tumorigenicity of AstroEGFR*2 astrocytes (Fig. 7b), validating the model in which the SMAD-dependent differentiating function of *Zfp423* is critical for its antigliomagenic properties.

## Discussion

HGG constitute an unmet challenge in oncology. Beyond the obvious difficulty linked to the anatomical site and the remarkable invasiveness of these tumours, two key problems have hampered progress so far: (i) the marked heterogeneity of these tumours, which implies the development of several subgroup-targeted approaches, and (ii) the virtual inaccessibility of early-stage lesions, with the attending inability to trace tumour history and define the critical gene circuits that drive initial establishment of the tumour. Notably, although many mouse models have proven invaluable in dissecting central aspects of this human disease, the experimental features of most of them precluded a thorough characterization of disease dynamics. This applies especially to the chromatin level of regulation, despite its emergence as a fundamental domain of perturbation that is raising major therapeutic hopes. Indeed, although cancer-specific chromatin maps, including for PRC2-dependent H3K27 trimethylation, are rapidly accumulating for both human and murine tumours, they most often describe endpoints of disease progression and are seldom related to transcriptional output in terms of mechanistically tested and functionally validated circuits.

Here we advance significantly our understanding of PRC2 contribution to gliomagenesis with an unbiased characterization of the PRC2-dependent circuits. Specifically, we combined the informative power of a mouse model featuring homogeneous cell populations of differential tumorigenic potential with a computational pipeline for the systematic integration of RNAseq and ChIPseq profiles with discovery tools for TF motif enrichment. This yielded two key insights. First, it enabled us to functionally partition the effect of PRC2 silencing, by showing that two critical developmental domains, one centred on the morphogenetic cues that enable migration and the other centred on dedifferentiation, were under direct PRC2 control. Importantly, both have been linked to core features of HGG, such as the marked propensity for invasiveness and the aberrant re-expression of elements of the pluripotency network^33^. Second, it led to the identification of a single TF, *Zfp423*, that was also under direct PRC2 control and accounted for a significant portion of the transcriptional dysregulation that underlies the transition from tumorigenic astrocytes to GPCs. Importantly, we found that *Zfp423* downregulation correlated with a worse prognosis, both in two independent mouse models and in two very large cohorts of human patients. The human homologue of *Zfp423*, *ZNF423*, had been previously identified as a positive prognostic factor for neuroblastoma^44^; hence, our results now extend the functional relevance of this gene to a wholly different class of tumours.

The modular architecture of *Zfp423* and ZNF423, whose Krüppel-like C2H2 zinc finger clusters mediate interaction with distinct transcriptional partners^62^, allowed us to mechanistically define the requirement of the BMP signalling axis for the anti-gliomagenic activity of *Zfp423*, as only the full-length protein but not its SMAD mutant form was able to prolong survival. This finding is particularly interesting in light of our results demonstrating PRC2-silencing of *Bmp4* in our primary tumour samples and of previous data on the aberrant recruitment of PRC2 to the *BMPR1B* receptor gene^28^, pointing to a synergic impact of Polycomb both on core members of the BMP signalling pathway and on one of their critical downstream effectors.

Finally, our findings also uncovered a significant degree of heterogeneity within *Ink4/Arf−/−*; EGFR* gliomagenesis, which we harnessed at the molecular level to define the mechanisms and context specificity of *Zfp423* antigliomagenic activity. As to the former, we found that *Ink4/Arf−/−*; EGFR* tumorigenic astrocytes, despite their overall similarity, display transcriptional specificities that are differentially retained in the gliomas they originate, suggesting that they represent differentially primed entry points of tumorigenesis. Consistently, this heterogeneity is carried through the primary gliomas, which encompass a relatively wide range of high-grade histopathological subtypes and also recapitulate, to different extents and for different parts, the signatures used to classify human HGG.

This transcriptomic dissection of glioma heterogeneity was functionally validated with the observation that *Zfp423* exerts the strongest antigliomagenic activity only in the context of the least tumour-primed astrocytes, in which it antagonizes, in an exquisitely SMAD signalling-dependent manner, a very significant portion of the gliomagenic programme that includes *Sox2*, a well-established glioma oncogene, and several other differentiation-promoting factors that are specifically downregulated in gliomas. However, the context specificity displayed in this experimental model did not prevent the definition of a general prognostic impact for *ZNF423* downregulation, which was confirmed in two large independent human cohorts. The context effect of *Zfp423* is consistent with accumulating evidence on the extreme heterogeneity that characterizes gliomas, with a growing number of TFs being shown to play critical roles but in highly selected cases, as in the instance of the six TFs that underlie transcriptomic changes uniquely in the mesenchymal subtype HGG^26^ or, even more strikingly, the *FGFR-TACC* oncogenic fusion that was identified in three patients from a large cohort of HGG patients^64^. Indeed, especially in light of the significant challenge posed by this remarkable heterogeneity, we believe that our approach of identifying candidate master regulators, along with their relevant networks, by comparing initiating cells with their corresponding tumours further advances the functional reclassification of gliomas on the basis of physiopathologically meaningful TF-centred pathways.

## Methods

### Human cells

Human GBM cell lines were obtained from Rossella Galli's lab (San Raffaele Scientific Institute, Milan, Italy) and were grown as described in ref. 65. Human astrocytes were obtained from ScienCell Research Laboratories (1810).

### Murine cell culture and manipulation

Primary astrocytes, GPCs and NPs were derived and cultured as described previously^66^,^67^,^43^ (see also Supplementary Methods). For the astrocyte cultures, we made use of a classic protocol first descbribed in 1980. Five- to 7-day-old pups were killed, each brain was removed from the head and transferred into a cell culture dish containing sterile Dulbecco's PBS (Lonza). Cerebral cortices were isolated from the rest of the brain and placed into a 1.5-ml tube containing 0.5 ml of astrocyte culture medium (15% North American fetal bovine serum (FBS) (Gibco), 2 mM glutamine, (Lonza), 0.6% glucose (Sigma), 100 U ml^−1^ potassium penicillin+100 U ml^−1^ streptomycin sulfate (Lonza) in basal medium Eagle's (Gibco)). They were mechanically dissociated with scissors and through pipetting; the dissociated tissue was plated into a T-25 flask and incubated in a humidified incubator at 37 °C with 5% CO_2_. Medium was changed 3 days after plating and every other day thereafter until day 9 of culture. Cells were passaged every 4–5 days starting from day 10 of culture at a 1:2 or 1:3 ratio. The cells were rinsed with Dulbecco's PBS and detached in trypsin–versene 1:10 (Sigma and Lonza, respectively); after detachment, the dissociation into single cells was achieved through pipetting. Trypsin was inactivated by adding FBS (Gibco, 1 volume per volume of trypsin) and cells were collected by centrifugation at 280 *g* for 5 min. The cell pellet was resuspended in fresh astrocyte medium and cells were plated in 6-well plates. EGFR*- or ZFP423/ZFP423dSBD-expressing retroviral particles were generated using the retrovirus producer cell line Phoenix-Eco. Established astrocyte cultures (after passage 2) were infected using the viral supernatant filtered through a 0.45-μm filter. Astrocytes were trypsinized as described before; 5 × 105 cells were resuspended in 3 ml of viral supernatant supplemented with 8 μg ml^−1^ Polybrene (Sigma) and plated in a well of a 6-well plate. The supernatant was removed and replaced with fresh supernatant at each new collection. Astrocytes were kept in culture for less than ten passages before being used for *in-vivo* experiments and next generation sequencing profiling.

GPC cultures were obtained and maintained as described in Pollard *et al.*^67^. Mice injected with tumorigenic astrocytes were killed at the onset of neurological symptoms. The tumour mass was dissected from the brain and a small piece was put in a 1.5-ml tube containing 0.5 ml of Accutase (Sigma). The tissue was mechanically dissociated with scissors and through pipetting, passed through a 70-μm cell strainer and single cell suspension was collected in a 50-ml tube. After centrifugation (300 *g* for 5 min), cells were resuspended in glioma-initiating cell medium (1 × B27, 1 × N2 (Gibco), 20 ng ml^−1^ of both EGF and murine fibroblast growth factor-2, 100 U ml^−1^ potassium penicillin+100 μg ml^−1^ streptomycin sulfate in neurobasal medium (Gibco)) and plated in one or two wells of a six-well plate, coated with laminin (Roche). Plates were kept in a humidified incubator 37 °C with 5% CO_2_; medium was changed 2 days after plating and every other day thereafter. Cells were passaged at 80–90% confluency at a ratio ranging from 1:3 to 1:6. They were detached with Accutase and dispersed into single cells by gentle pipetting; after centrifugation at 300 *g* for 5 min, cells were resuspended in glioma-initiating cell medium and plated as described above. GPCs were kept in culture for less than ten passages before being used for *in-vivo* experiments and next generation sequencing profiling. Differentiation was performed according to the protocol published in the same paper. In particular, astrocytic differentiation was performed by adding FBS or BMP4 to the culturing medium, whereas neuronal differentiation was achieved through growth factor withdrawal. The same protocol was used for the differentiation of embryonic neural precursors.

EGFR*, Pdgf-β, Zfp423 or Zfp423DSBD retroviral particles were generated using the retrovirus producer cell line Phoenix-Eco. Supernatant was collected and used directly or after ultracentrifugation. Differentiation of NP was performed as described in ref. 67.

### *In vivo* experiments

Experiments involving animals were carried out in accordance with the Italian Laws (D.lgs. 26/2014), which enforces Dir. 2010/63/EU (Directive 2010/63/EU of the European Parliament and of the Council of 22 September 2010, on the protection of animals used for scientific purposes). Specifically, the project was notified to the Ministry of Health before the implementation of the current legislation. As the new legislation does not apply to projects approved before its enactment, for such projects only a notification of the experiments to the Ministry of Health was required (in accordance with the D.L.vo 116/92 (and following additions), which enforced EU 86/609 Directive), and this requirement was dutifully fulfilled.

Tumour formation was induced by stereotaxical injection of 5 × 10^5^ AstroEGFR* or GPCs into the caudate of Cd1-Nude HO, 5-week-old female mice (coordinates: 0.7 mm back, 3 mm left and 3.5 mm deep from the bregma). Brains were isolated at the onset of disease symptoms and fixed in 4% buffered formalin. Samples were paraffin embedded, sectioned, stained for haematoxylin and eosin, and histopathologically classified by pathologists.

### RNA and protein extraction

RNA was extracted from all the lines using the RNeasy Mini Plus Kit (Qiagen) according to the manufacturer's specifications. Quality and concentration of RNA was assessed using a NanoDropSpectrophotometer (NanoDrop Technologies).

Proteins were extracted as follows: cells were harvested from the plate and centrifuged at 300 *g* for 5 min, then washed in PBS and lysed in RIPA buffer plus protease inhibitors cocktail (Sigma). Lysates were sonicated using the Bioruptor Sonication System (UCD200) for three cycles of 30 s with 60 s pause at high power. Lysates were centrifuged at 13,000 *g* for 15 min and supernatants were transferred to a new tube. Protein quantification was performed using the Bradford protein assay (BioRad) following the manufacturer's instructions.

### Complementary DNA preparation and qPCR

Retrotranscribed cDNAs have been obtained from 1 μg of total DNA-depleted RNA using the superscript VILO retrotranscription kit from Life Technologies according to the manufacturer's instructions.

For quantitative reverse transcriptase–PCR (qRT–PCR) analysis, a total amount of cDNA corresponding to 5 ng of starting RNA has been used for each reaction. FAST SYBR green master mix from Life Technologies and 10 μM primer pairs have been used. The qPCR reactions have been performed on an Applied Biosystems 7500 Real-Time PCR machine following the standard amplification protocol (primer list and Taqman Assays ID provided in Supplementary Tables 1 and 2).

### RNAseq and ChIPseq

Library preparation for RNA sequencing was performed using RiboZero and Single Stranded kits (Illumina) according to the manufacturer's instructions. Validation of RNAseq results has been performed through qRT–PCR analysis.

For ChIPseq, cells were harvested and resuspended in PBS containing 1% formaldehyde for fixation and quenched with 125 mM glycine. Cells were lysed and sonicated using the Covaris Sonicator, to generate 250 bp DNA fragments. Chromatin was incubated overnight at 4 °C with the antibodies (H3K27me3 (Cell Signalling) and H3 total (Abcam)) and recovered the day after using Dynabeads Protein G (Life Technologies). Immunocomplexes were eluted, de-cross-linked and purified using Qiaquick PCR Columns (Qiagen) according to the manufacturer's instructions. DNA libraries were prepared according to ChIPseq Sample Preparation Guide (Illumina) and sequenced on an Illumina GA-II or HiSeq 2000 platform. Validation of ChIPseq results has been performed through qPCR analysis: 5 ng DNA have been used for each reaction. FAST SYBR green master mix from Life Technologies and 10 μM primer pairs have been used. The qPCR reactions have been performed on an Applied Biosystems 7500 Real-Time PCR machine following the standard amplification protocol. Primer lists for qRT–PCR and ChIP–qPCR are provided in Supplementary Tables 1 and 3. A detailed description of computational analysis is available as Supplementary Methods. Gene lists from ChIPseq and RNAseq analysis are available as Supplementary Data 1. IPA analyses are available as Supplementary Data 2. ChIPseq and RNAseq data have been deposited in the GEO database (GSE76292).

## Additional information

**How to cite this article:** Signaroldi, E. *et al.* Polycomb dysregulation in gliomagenesis targets a *Zfp423*-dependent differentiation network. *Nat. Commun.* 7:10753 doi: 10.1038/ncomms10753 (2016).

## Supplementary Material

Supplementary Information Supplementary Figures 1-6, Supplementary Tables 1-3, Supplementary Methods and Supplementary References

Supplementary Data 1 Gene lists from ChIPseq and RNAseq analysis

Supplementary Data 2 Gene lists from IPA analysis.

## References

[b1] Fragola G. *et al.* Cell reprogramming requires silencing of a core subset of polycomb targets. PLoS Genet. 9, e1003292 (2013). 23468641 10.1371/journal.pgen.1003292 PMC3585017

[b2] Laugesen A. & Helin K. Chromatin repressive complexes in stem cells, development, and cancer. Cell Stem Cell 14, 735–751 (2014). 24905164 10.1016/j.stem.2014.05.006

[b3] Riising E. M. *et al.* Gene silencing triggers polycomb repressive complex 2 recruitment to CpG islands genome wide. Mol. Cell 55, 347–360 (2014). 24999238 10.1016/j.molcel.2014.06.005

[b4] Margueron R. & Reinberg D. The Polycomb complex PRC2 and its mark in life. Nature 469, 343–349 (2011). 21248841 10.1038/nature09784 PMC3760771

[b5] Ohm J. E. *et al.* A stem cell-like chromatin pattern may predispose tumour suppressor genes to DNA hypermethylation and heritable silencing. Nat. Genet. 39, 237–242 (2007). 17211412 10.1038/ng1972 PMC2744394

[b6] Schlesinger Y. *et al.* Polycomb-mediated methylation on Lys27 of histone H3 pre-marks genes for de novo methylation in cancer. Nat. Genet. 39, 232–236 (2007). 17200670 10.1038/ng1950

[b7] Widschwendter M. *et al.* Epigenetic stem cell signature in cancer. Nat. Genet. 39, 157–158 (2007). 17200673 10.1038/ng1941

[b8] Marchesi I., Fiorentino F. P., Rizzolio F., Giordano A. & Bagella L. The ablation of EZH2 uncovers its crucial role in rhabdomyosarcoma formation. Cell Cycle 11, 3828–3836 (2012). 22983009 10.4161/cc.22025 PMC3495825

[b9] Chen T. & Dent S. Y. Chromatin modifiers and remodellers: regulators of cellular differentiation. Nat. Rev. Genet. 15, 93–106 (2014). 24366184 10.1038/nrg3607 PMC3999985

[b10] Chaffer C. L. *et al.* Poised chromatin at the ZEB1 promoter enables breast cancer cell plasticity and enhances tumorigenicity. Cell 154, 61–74 (2013). 23827675 10.1016/j.cell.2013.06.005 PMC4015106

[b11] Bracken A. P. *et al.* The Polycomb group proteins bind throughout the INK4A-ARF locus and are disassociated in senescent cells. Genes Dev. 21, 525–530 (2007). 17344414 10.1101/gad.415507 PMC1820894

[b12] Lu C. *et al.* Regulation of tumour angiogenesis by EZH2. Cancer Cell 18, 185–197 (2010). 20708159 10.1016/j.ccr.2010.06.016 PMC2923653

[b13] Piunti A. *et al.* Polycomb proteins control proliferation and transformation independently of cell cycle checkpoints by regulating DNA replication. Nat. Commun. 5, 3649 (2014). 24728135 10.1038/ncomms4649 PMC3996544

[b14] Caganova M. *et al.* Germinal center dysregulation by histone methyltransferase EZH2 promotes lymphomagenesis. J. Clin. Invest. 123, 5009–5022 (2013). 24200695 10.1172/JCI70626 PMC3859423

[b15] McCabe M. T. *et al.* EZH2 inhibition as a therapeutic strategy for lymphoma with EZH2-activating mutations. Nature 492, 108–112 (2012). 23051747 10.1038/nature11606

[b16] Bracken A. P. *et al.* EZH2 is downstream of the pRB-E2F pathway, essential for proliferation and amplified in cancer. EMBO J. 22, 5323–5335 (2003). 14532106 10.1093/emboj/cdg542 PMC213796

[b17] Ernst T. *et al.* Inactivating mutations of the histone methyltransferase gene EZH2 in myeloid disorders. Nat. Genet. 42, 722–726 (2010). 20601953 10.1038/ng.621

[b18] Lee W. *et al.* PRC2 is recurrently inactivated through EED or SUZ12 loss in malignant peripheral nerve sheath tumours. Nat. Genet. 46, 1227–1232 (2014). 25240281 10.1038/ng.3095 PMC4249650

[b19] Schwartzentruber J. *et al.* Driver mutations in histone H3.3 and chromatin remodelling genes in paediatric glioblastoma. Nature 482, 226–231 (2012). 22286061 10.1038/nature10833

[b20] Wu G. *et al.* Somatic histone H3 alterations in pediatric diffuse intrinsic pontine gliomas and non-brainstem glioblastomas. Nat. Genet. 44, 251–253 (2012). 22286216 10.1038/ng.1102 PMC3288377

[b21] de Vries N. A. *et al.* Prolonged Ezh2 depletion in glioblastoma causes a robust switch in cell fate resulting in tumour progression. Cell Rep. 10, 383–397 (2015). 10.1016/j.celrep.2014.12.028 25600873

[b22] Koppens M. & van Lohuizen M. Context-dependent actions of Polycomb repressors in cancer. Oncogene 44, 251–253 (2015). 10.1038/onc.2015.195 26050622

[b23] Steffen P. A. & Ringrose L. What are memories made of? How Polycomb and Trithorax proteins mediate epigenetic memory. Nat. Rev. Mol. Cell Biol. 15, 340–356 (2014). 24755934 10.1038/nrm3789

[b24] Phillips H. S. *et al.* Molecular subclasses of high-grade glioma predict prognosis, delineate a pattern of disease progression, and resemble stages in neurogenesis. Cancer Cell 9, 157–173 (2006). 16530701 10.1016/j.ccr.2006.02.019

[b25] Verhaak R. G. *et al.* Integrated genomic analysis identifies clinically relevant subtypes of glioblastoma characterized by abnormalities in PDGFRA, IDH1, EGFR, and NF1. Cancer Cell 17, 98–110 (2010). 20129251 10.1016/j.ccr.2009.12.020 PMC2818769

[b26] Carro M. S. *et al.* The transcriptional network for mesenchymal transformation of brain tumours. Nature 463, 318–325 (2010). 20032975 10.1038/nature08712 PMC4011561

[b27] Suva M. L. *et al.* Reconstructing and reprogramming the tumour-propagating potential of glioblastoma stem-like cells. Cell 157, 580–594 (2014). 24726434 10.1016/j.cell.2014.02.030 PMC4004670

[b28] Lee J. *et al.* Epigenetic-mediated dysfunction of the bone morphogenetic protein pathway inhibits differentiation of glioblastoma-initiating cells. Cancer Cell 13, 69–80 (2008). 18167341 10.1016/j.ccr.2007.12.005 PMC2835498

[b29] Orzan F. *et al.* Enhancer of Zeste 2 (EZH2) is up-regulated in malignant gliomas and in glioma stem-like cells. Neuropathol. Appl. Neurobiol. 37, 381–394 (2011). 20946108 10.1111/j.1365-2990.2010.01132.x

[b30] Bruggeman S. W. *et al.* Bmi1 controls tumour development in an Ink4a/Arf-independent manner in a mouse model for glioma. Cancer Cell 12, 328–341 (2007). 17936558 10.1016/j.ccr.2007.08.032

[b31] Gargiulo G. *et al.* *In vivo* RNAi screen for BMI1 targets identifies TGF-beta/BMP-ER stress pathways as key regulators of neural- and malignant glioma-stem cell homeostasis. Cancer Cell 23, 660–676 (2013). 23680149 10.1016/j.ccr.2013.03.030

[b32] Bachoo R. M. *et al.* Epidermal growth factor receptor and Ink4a/Arf: convergent mechanisms governing terminal differentiation and transformation along the neural stem cell to astrocyte axis. Cancer Cell 1, 269–277 (2002). 12086863 10.1016/s1535-6108(02)00046-6

[b33] Friedmann-Morvinski D. & Verma I. M. Dedifferentiation and reprogramming: origins of cancer stem cells. EMBO Rep. 15, 244–253 (2014). 24531722 10.1002/embr.201338254 PMC3989690

[b34] Vogel T., Ahrens S., Buttner N. & Krieglstein K. Transforming growth factor beta promotes neuronal cell fate of mouse cortical and hippocampal progenitors in vitro and in vivo: identification of Nedd9 as an essential signalling component. Cereb. Cortex 20, 661–671 (2010). 19587023 10.1093/cercor/bhp134 PMC2820705

[b35] Gomes W. A., Mehler M. F. & Kessler J. A. Transgenic overexpression of BMP4 increases astroglial and decreases oligodendroglial lineage commitment. Dev. Biol. 255, 164–177 (2003). 12618141 10.1016/s0012-1606(02)00037-4

[b36] Ozair M. Z., Noggle S., Warmflash A., Krzyspiak J. E. & Brivanlou A. H. SMAD7 directly converts human embryonic stem cells to telencephalic fate by a default mechanism. Stem Cells 31, 35–47 (2013). 23034881 10.1002/stem.1246 PMC4103884

[b37] Mohn F. *et al.* Lineage-specific polycomb targets and de novo DNA methylation define restriction and potential of neuronal progenitors. Mol. Cell 30, 755–766 (2008). 18514006 10.1016/j.molcel.2008.05.007

[b38] Jiang C. & Pugh B. F. A compiled and systematic reference map of nucleosome positions across the Saccharomyces cerevisiae genome. Genome Biol. 10, R109 (2009). 19814794 10.1186/gb-2009-10-10-r109 PMC2784324

[b39] Brose K. *et al.* Slit proteins bind Robo receptors and have an evolutionarily conserved role in repulsive axon guidance. Cell 96, 795–806 (1999). 10102268 10.1016/s0092-8674(00)80590-5

[b40] Li H. S. *et al.* Vertebrate slit, a secreted ligand for the transmembrane protein roundabout, is a repellent for olfactory bulb axons. Cell 96, 807–818 (1999). 10102269 10.1016/s0092-8674(00)80591-7

[b41] Dallol A. *et al.* Frequent epigenetic inactivation of the SLIT2 gene in gliomas. Oncogene 22, 4611–4616 (2003). 12881718 10.1038/sj.onc.1206687

[b42] Yiin J. J. *et al.* Slit2 inhibits glioma cell invasion in the brain by suppression of Cdc42 activity. Neuro Oncol. 11, 779–789 (2009). 20008733 10.1215/15228517-2008-017 PMC2802398

[b43] Appolloni I. *et al.* PDGF-B induces a homogeneous class of oligodendrogliomas from embryonic neural progenitors. Int. J. Cancer 124, 2251–2259 (2009). 19165863 10.1002/ijc.24206

[b44] Huang S. *et al.* ZNF423 is critically required for retinoic acid-induced differentiation and is a marker of neuroblastoma outcome. Cancer Cell 15, 328–340 (2009). 19345331 10.1016/j.ccr.2009.02.023 PMC2693316

[b45] Mazzoleni S. *et al.* Epidermal growth factor receptor expression identifies functionally and molecularly distinct tumour-initiating cells in human glioblastoma multiforme and is required for gliomagenesis. Cancer Res. 70, 7500–7513 (2010). 20858720 10.1158/0008-5472.CAN-10-2353

[b46] Madhavan S. *et al.* Rembrandt: helping personalized medicine become a reality through integrative translational research. Mol. Cancer Res. 7, 157–167 (2009). 19208739 10.1158/1541-7786.MCR-08-0435 PMC2645472

[b47] Gupta R. K. *et al.* Transcriptional control of preadipocyte determination by Zfp423. Nature 464, 619–623 (2010). 20200519 10.1038/nature08816 PMC2845731

[b48] Montojo J. *et al.* GeneMANIA Cytoscape plugin: fast gene function predictions on the desktop. Bioinformatics 26, 2927–2928 (2010). 20926419 10.1093/bioinformatics/btq562 PMC2971582

[b49] Loddo M. *et al.* Pregnancy-associated plasma protein A regulates mitosis and is epigenetically silenced in breast cancer. J. Pathol. 233, 344–356 (2014). 24931331 10.1002/path.4393

[b50] Fougere M. *et al.* NFAT3 transcription factor inhibits breast cancer cell motility by targeting the Lipocalin 2 gene. Oncogene 29, 2292–2301 (2010). 20101218 10.1038/onc.2009.499

[b51] Kaneda A. *et al.* Lysyl oxidase is a tumour suppressor gene inactivated by methylation and loss of heterozygosity in human gastric cancers. Cancer Res. 64, 6410–6415 (2004). 15374948 10.1158/0008-5472.CAN-04-1543

[b52] Favaro R. *et al.* Sox2 is required to maintain cancer stem cells in a mouse model of high-grade oligodendroglioma. Cancer Res. 74, 1833–1844 (2014). 24599129 10.1158/0008-5472.CAN-13-1942

[b53] de la Rocha A. M., Sampron N., Alonso M. M. & Matheu A. Role of SOX family of transcription factors in central nervous system tumours. Am. J. Cancer Res. 4, 312–324 (2014). 25057435 PMC4106650

[b54] Huang Y., Das A. K., Yang Q. Y., Zhu M. J. & Du M. Zfp423 promotes adipogenic differentiation of bovine stromal vascular cells. PLoS ONE 7, e47496 (2012). 23071815 10.1371/journal.pone.0047496 PMC3468566

[b55] Alcaraz W. A. *et al.* Zfp423 controls proliferation and differentiation of neural precursors in cerebellar vermis formation. Proc. Natl Acad. Sci. USA 103, 19424–19429 (2006). 17151198 10.1073/pnas.0609184103 PMC1748242

[b56] Cheng L. E., Zhang J. & Reed R. R. The transcription factor Zfp423/OAZ is required for cerebellar development and CNS midline patterning. Dev. Biol. 307, 43–52 (2007). 17524391 10.1016/j.ydbio.2007.04.005 PMC2866529

[b57] Warming S., Rachel R. A., Jenkins N. A. & Copeland N. G. Zfp423 is required for normal cerebellar development. Mol. Cell. Biol. 26, 6913–6922 (2006). 16943432 10.1128/MCB.02255-05 PMC1592861

[b58] Cheng L. E. & Reed R. R. Zfp423/OAZ participates in a developmental switch during olfactory neurogenesis. Neuron 54, 547–557 (2007). 17521568 10.1016/j.neuron.2007.04.029 PMC2866517

[b59] Roby Y. A. *et al.* Zfp423/OAZ mutation reveals the importance of Olf/EBF transcription activity in olfactory neuronal maturation. J. Neurosci. 32, 13679–13688a (2012). 23035080 10.1523/JNEUROSCI.6190-11.2012 PMC3483594

[b60] Ziller M. J. *et al.* Dissecting neural differentiation regulatory networks through epigenetic footprinting. Nature 518, 355–359 (2015). 25533951 10.1038/nature13990 PMC4336237

[b61] Cahoy J. D. *et al.* A transcriptome database for astrocytes, neurons, and oligodendrocytes: a new resource for understanding brain development and function. J. Neurosci. 28, 264–278 (2008). 18171944 10.1523/JNEUROSCI.4178-07.2008 PMC6671143

[b62] Hata A. *et al.* OAZ uses distinct DNA- and protein-binding zinc fingers in separate BMP-Smad and Olf signalling pathways. Cell 100, 229–240 (2000). 10660046 10.1016/s0092-8674(00)81561-5

[b63] Masserdotti G. *et al.* ZFP423 coordinates Notch and bone morphogenetic protein signalling, selectively up-regulating Hes5 gene expression. J. Biol. Chem. 285, 30814–30824 (2010). 20547764 10.1074/jbc.M110.142869 PMC2945575

[b64] Di Stefano A. L. *et al.* Detection, characterization, and inhibition of FGFR-TACC fusions in IDH wild-type glioma. Clin. Cancer Res. 21, 3307–3317 (2015). 25609060 10.1158/1078-0432.CCR-14-2199 PMC4506218

[b65] Galli R. *et al.* Isolation and characterization of tumourigenic, stem-like neural precursors from human glioblastoma. Cancer Res. 64, 7011–7021 (2004). 15466194 10.1158/0008-5472.CAN-04-1364

[b66] McCarthy K. D. & de Vellis J. Preparation of separate astroglial and oligodendroglial cell cultures from rat cerebral tissue. J. Cell Biol. 85, 890–902 (1980). 6248568 10.1083/jcb.85.3.890 PMC2111442

[b67] Pollard S. M. *et al.* Glioma stem cell lines expanded in adherent culture have tumour-specific phenotypes and are suitable for chemical and genetic screens. Cell Stem Cell 4, 568–580 (2009). 19497285 10.1016/j.stem.2009.03.014

