# Supplementary material for: Polycomb dysregulation in gliomagenesis targets a Zfp423-dependent differentiation network
Source: Nat Commun. 2016 Feb 29;7:10753. doi: 10.1038/ncomms10753 (PMC4773478; doi:10.1038/ncomms10753)
Supplement: Supplementary Information — Supplementary Figures 1-6, Supplementary Tables 1-3, Supplementary Methods and Supplementary References [file ncomms10753-s1.pdf]

Supplementary Figures

Supplementary Figure 1

a

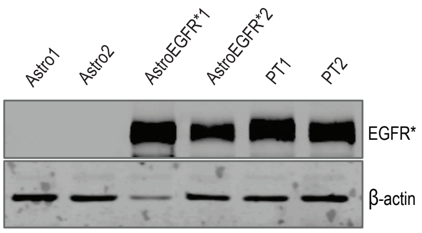

b

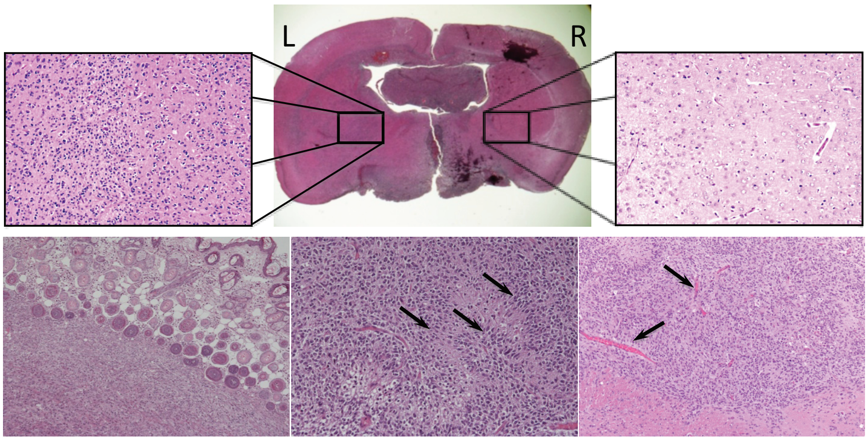

c

Classification	Percentage
Oligoastrocitoma	11%
Gliosarcoma	18,5%
Oligodendroglioma	26%
Anaplastic Astrocytoma	7,5%
GBM	7,5%
Others	29,5%

Malignancy grade	Percentage
I	0
II	16%
III	42%
IV	42%

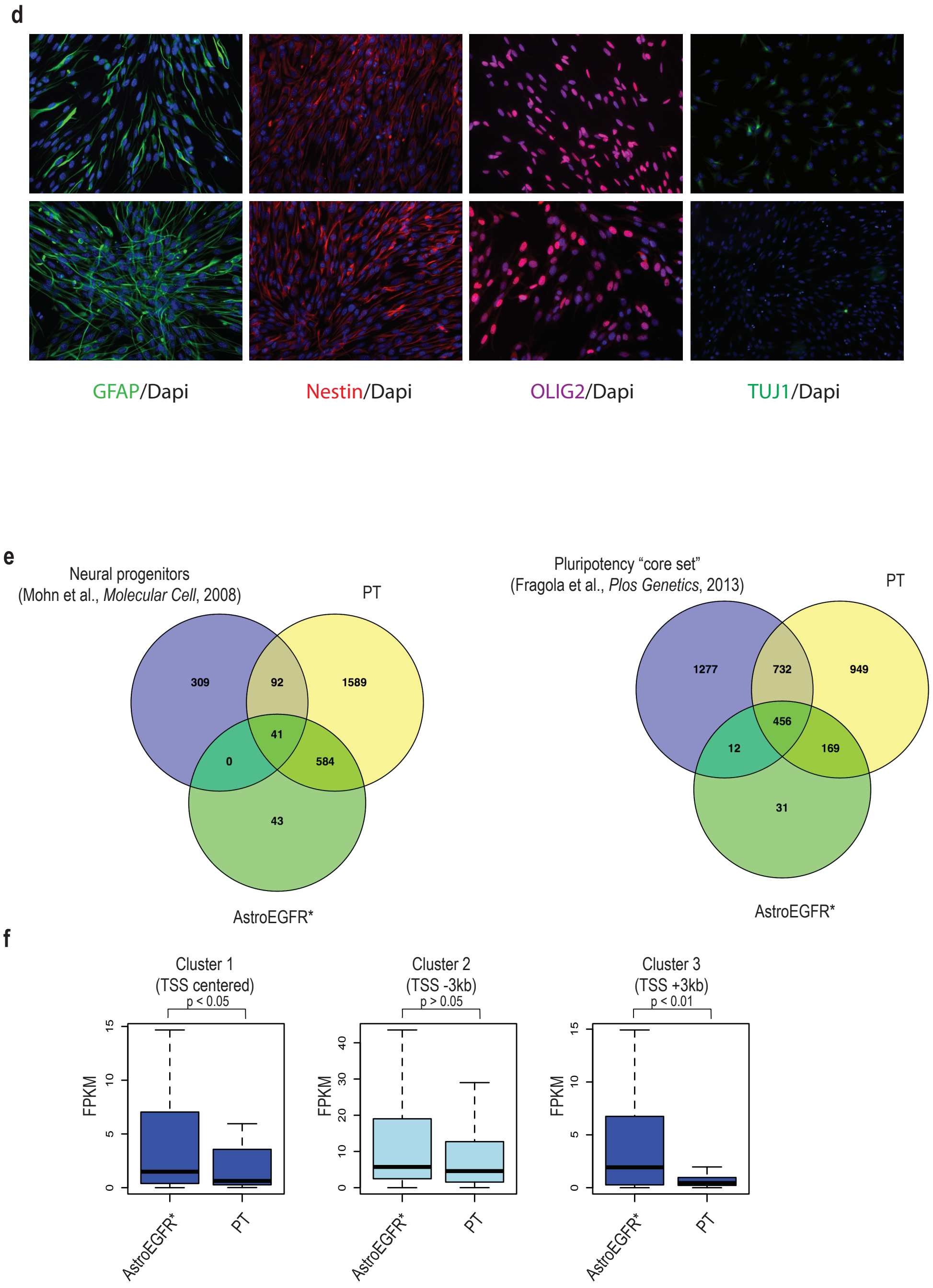

Supplementary Figure 1 | (a) Detection of EGFR* by Western blot in the different cell populations. β-actin was used as loading control. **(b)** Histological analysis of the tumors. At a macroscopic level, the injected hemisphere (left, L) appears enlarged compared to the right counterpart. In the close-ups a comparison is made between the left and the right parenchyma, the first one showing hypercellularity and a complete invasion by tumor cells. Lower panels, from left to right: subcutaneous layer invasion; pseudopalisade necrosis (indicated by arrows); new vessels formed during tumor development (indicated by arrows). **(c)** Histopathological classification of the tumors according to the World Health Organization system. Left table, histolgical classification of tumors; right table, malignancy grade were defined for all categories except "Others". **(d)** Cells derived from primary tumors (i.e. glioma propagting cells, GPC) were

stained for well defined glioma markers such as GFAP, Nestin and OLIG2. Staining for Tuj1 shows the absence of contaminating neurons in the cultures. (**e**) Left, Venn diagram showing the overlap between H3K27me3 target genes in AstroEGFR*, PT and neural precursors (as described in Mohn et al., 2008). The p-value associated with the specific overlap between H3K27me3 target genes in PT and neural precursors (i.e. 92 genes) is statistically significant ($p<0.001$, hypergeometric test). Right, Venn diagram showing the overlap between H3K27me3 target genes in AstroEGFR*, PT and the subset of Polycomb targets whose silencing is required for cell reprogramming as we previously identified in Fragola et al., 2013. (**f**) Box plots representing differences in gene expression for the *de novo* H3K27me3 target genes in PT. Cluster1 represents genes that acquired the mark preferentially on the TSS; Cluster2 represents genes that acquired the mark preferentially upstream (-3Kb) to the TSS; Cluster3 represents genes that acquired the mark preferentially downstream (+3Kb) to the TSS. P-values were computed with Mann-Whitney test.

Supplementary Figure 2

a

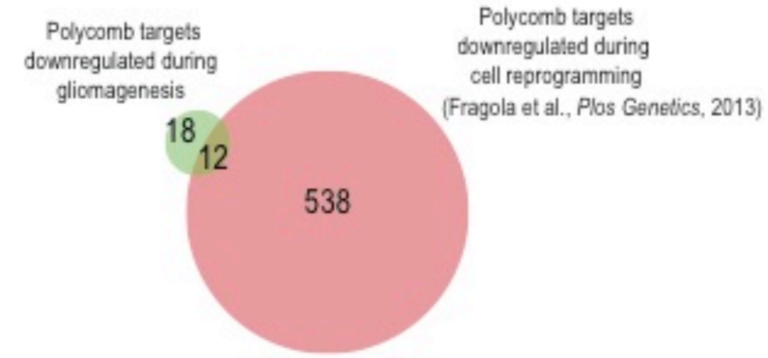

b

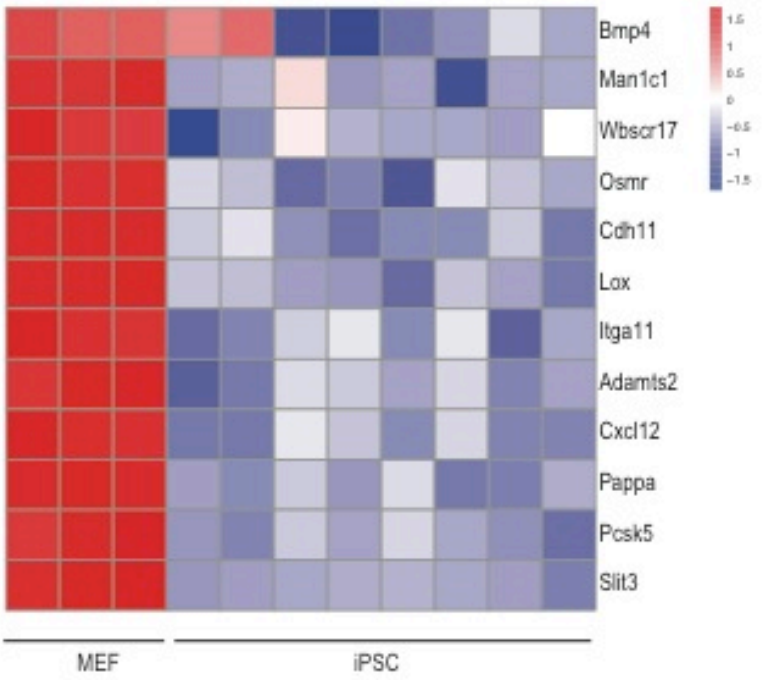

Supplementary Figure 2 | (**a**) Venn diagram displaying the overlap between the Polycomb targets downregulated during gliomagenesis and the Polycomb targets downregulated during cell reprogramming. The significance of the overlap was computed using the hypergeometric test, p=3.7e-13. (**b**) Heatmap showing the expression level of the 12 overlapping genes from (**a**) in mouse embryonic fibroblasts (MEF) and induced pluripotent stem cells (iPSC) from Fragola et *al*., Plos Genetics, 2013.

Supplementary Figure 3

a

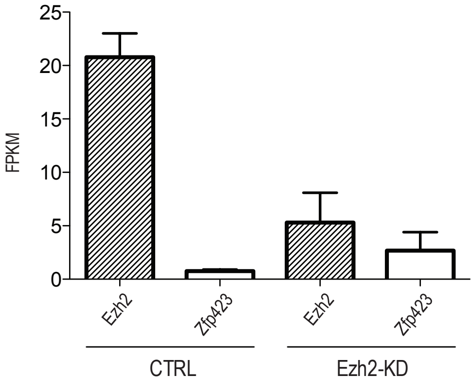

Supplementary Figure 3 | (a) Analysis of *Zfp423* expression in a tumor model in which the catalytic subunit of the PRC2, *Ezh2*, is knocked-down upon doxycycline administration. In control samples, *Zfp423* expression is lower compared to *Ezh2*-knock-down samples. Bars represents average FPKM for each gene, error bars represent the standard deviation (SD) of the biological replicates.

Supplementary Figure 4

a

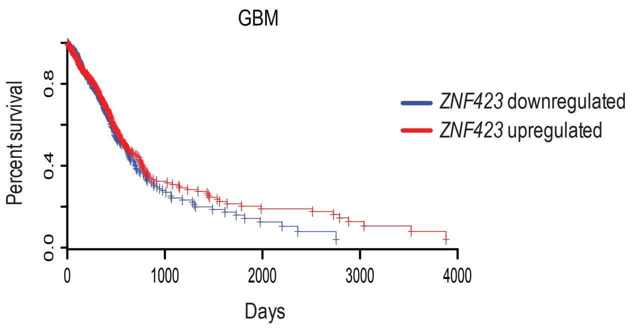

b

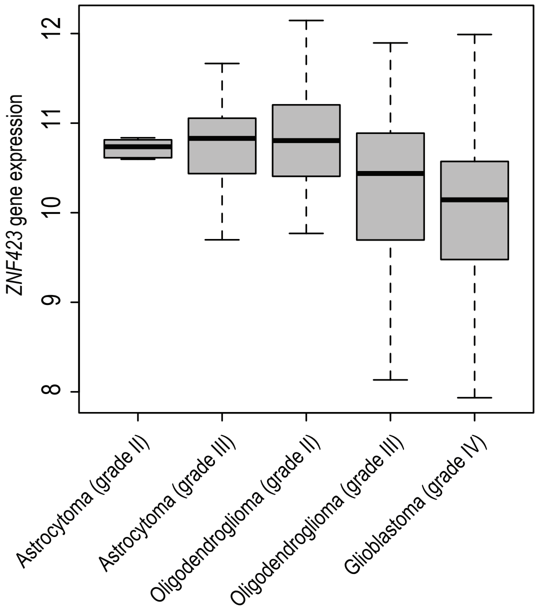

Supplementary Figure 4 | (a) Kaplan-Meyer analysis of survival in GBM patients with high (red, n=202) or low (blue, n=209) *ZNF423* expression levels. Data downloaded from TCGA, p-value (p=0.21) was computed by log-rank test (Mantel-Cox). (**b**) Boxplots represent expression levels of *ZNF423* in different samples grouped according to histopathological classification. Data downloaded from Gene Expression Omnibus (GEO ID = GSE4290).

Supplementary Figure 5

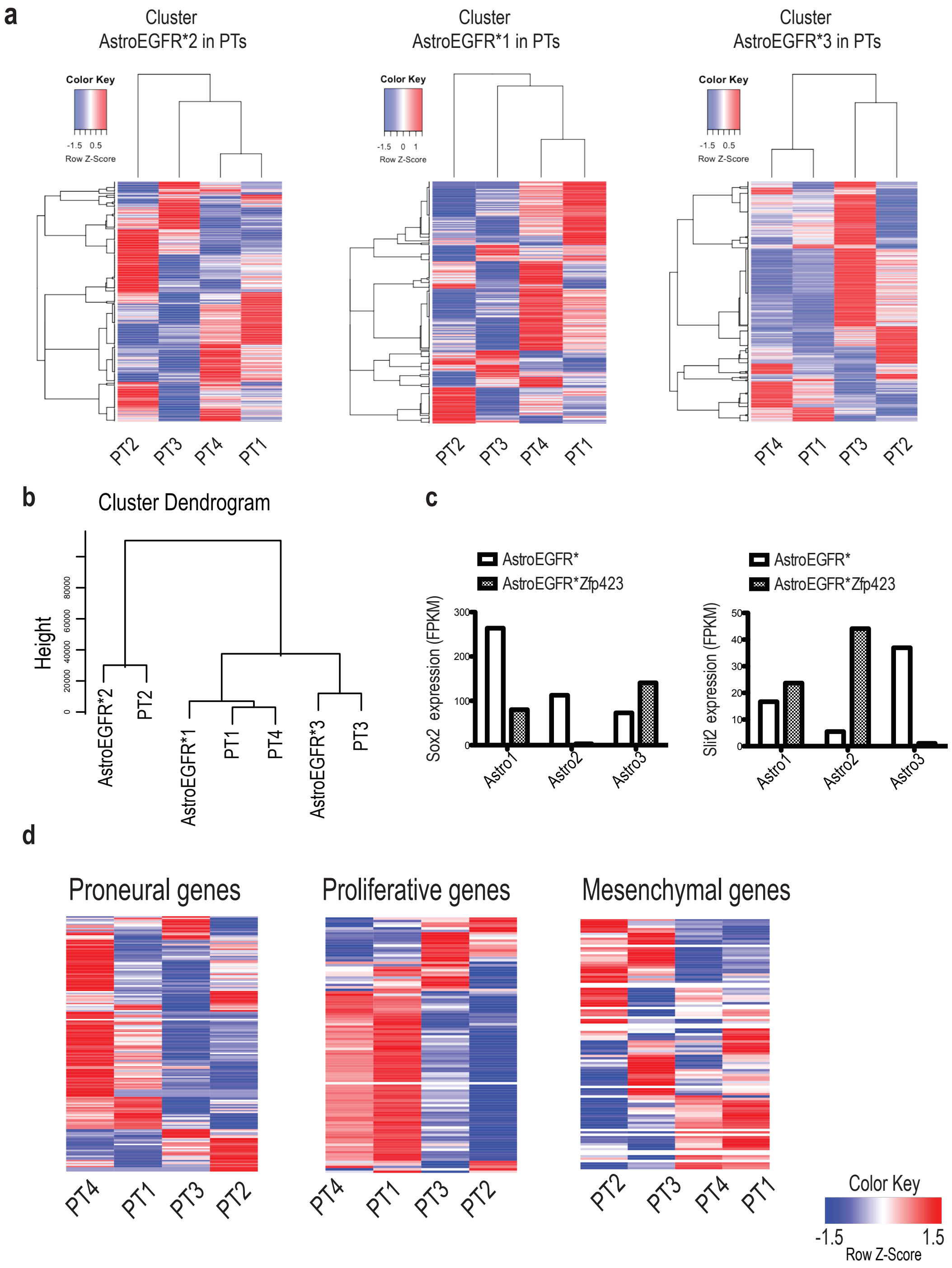

Supplementary Figure 5 | (**a**) Heatmaps and unsupervised cluster of transcriptomes from different PT on the basis of the AstroEGFR* cluster-specific genes depicted in Fig. 6b. (**b**) Whole transcriptome unsupervised cluster analysis of the three independent AstroEGFR* batches and their respective PT. (**c**) Barplots showing the differences in gene expression of selected genes that are differentially expressed upon *Zfp423* overexpression in the three batches of tumorigenic astrocytes. (**d**) Heatmaps and unsupervised clusters of the genes from our cohort of primary murine gliomas that are orthologous to the genes associated with the Proneural, Proliferative and Mesenchymal subtypes of human gliomas, as defined by Phillips et al., *Cancer Cell*, 2006.

Supplementary Figure 6

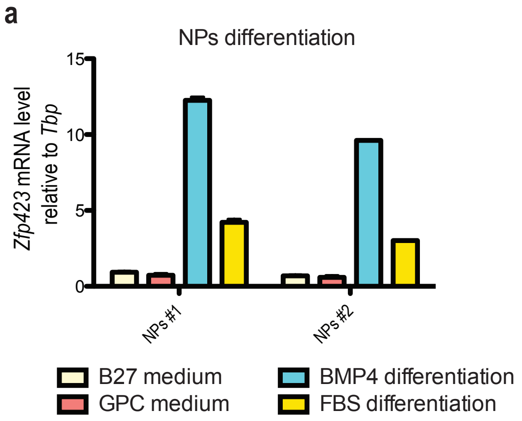

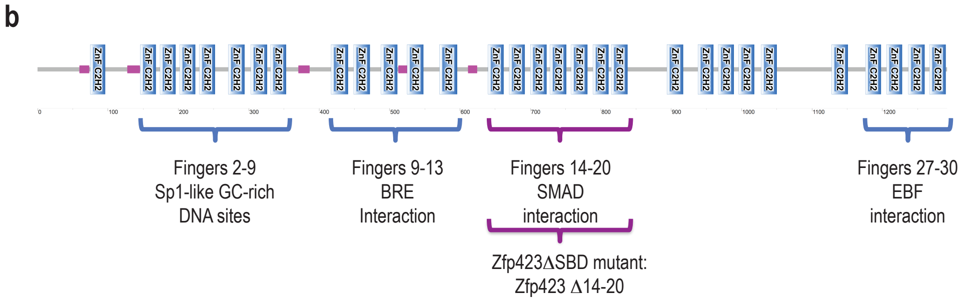

Supplementary Figure 6 | (**a**) Differentiation of neural precursors (NP) isolated from E15 embryos. *Zfp423* expression is increased in NP subjected to astrocytic differentiation, with the greatest induction achieved upon BMP4 administration. Data are represented as dCt (log_2 scale) relative to *Tbp*, error bars represent the standard deviation (SD) of the technical replicates. (**b**) ZFP423 motifs. The protein contains 30 Kruppel-like C_2H_2 zinc fingers grouped in clusters with different functional roles as mediators of distinct signaling pathways, as indicated by braces. The *Zfp423*ΔSBD mutant lacks the zinc fingers from 14 to 20.

Supplementary Tables

Supplementary Table 1

**Primers used in qRT-PCR**		
Gene	Primer Fwd	Primer Rev
Slit2	GTATCCCTCCACGAACCTTTG	TCACAGTAAAGAGGGTTGGC
Slit3	ATCTTCGAGTCTTGCATCTGG	TGGAAAAGCAGTTCTGGGAG
Bmp4	GAGGAGTTCCATCACGAAGA	GCTCTGCCGAGGAGATCA
Zfp423	ATCGGTGAAAGTTGAAGAGGG	ACTTGTCACGCTGTTCCTG
Tbp	TCCTGTGCACACCATTTTTC	CTGGAATTGTACCGCAGCTT
ZNF423	CACAGTGCCCTCAGAAGTTC	GGATGTAATGTTCAAATGGCCC
TBP	CACATCACAGCTCCCCACC	TGCACAGGAGCCAAGAGTGAA

Supplementary Table 1. Table contains primer list for qRT-PCR.

Supplementary Table 2

**Taqman® Applied Biosystem chemistry**	
Gene	Assay
Sox2	mm00488369_s1
Tbp	mm00446973_m1

Supplementary Table 2. Table contains Taqman® Assays ID.

Supplementary Table 3

**Primers used in ChIP-qPCR**		
Gene	Primer Fwd	Primer Rev
Slit2 - H3K27me3 positive region	TCGCTGGGGTTAGTGTTGTC	TCACAGTCTCTCGGTGTTGC
Slit2 - H3K27me3 negative region	GATCACTCGGAGTCGGGTTT	GCAGGAATCAGCACAAGCAG
Zfp423 - H3K27me3 positive region	CCCCGAGATTTATCCACGCA	CGAACTAGGCGCGAGAGTTA
Zfp423 - H3K27me3 negative region	GAGCCAAGATGGGCCAAGAT	GGGCCTAGACGTTTCCTTCC

Supplementary Table 3. Table contains primer list for ChIP-qPCR.

Supplementary Methods

Computational analysis

RNAseq analysis: RNASeq data were processed using tophat, cufflink/cuffidiff pipeline [1]. Reads were aligned to mouse reference genome (mm9) using TopHat1.4. Quantification of reads was performed with Cufflinks 2.2.1 using sequence-bias and multi-read corrections. Differential gene expression between AstroEGFR* and PT was estimated using Cufflinks 2.2.1 using "per-condition" dispersion models, and only the genes with a q-value <0.05 were considered as differentially expressed. For the comparison between AstroEGFR* and AstroEGFR* *Zfp423* we used the "blind" dispersion model. Only the top 500 DEGs were interrogated with Gene Mania database[2] to reconstruct a co-expression based network centered around *Zfp423* in each AstroEGFR*.
ChIPseq analysis: Reads were aligned to the mm9 genome using Bowtie[3] v.0.12.9 allowing up to two mismatches per read. Enriched regions for H3K27me3 were identified using MACS[4] 1.4.1 by disabling the shifting model and using a stringent p-value threshold of 1e-7. Target genes were identified as genes with an enriched region for H3K27me3 in the region that spans ±3kb around their transcription start site. Signal tracks were generated using IGVtools and visualized with IGV[5] using the "Normalize Coverage Data" option. Heatmaps data matrix files were generated using HOMER[6]. Clustering and visualization were performed with Cluster 3.0[7] and Java Treeview[8] respectively. Functional enrichment analysis for canonical pathways was performed with Ingenuity Pathway Analysis (IPA, QIAGEN Redwood City, www.qiagen.com/ingenuity). Gene set enrichment analysis was performed using GSEA software[9] from http://www.broadinstitute.org/gsea/index.jsp
Transcription factor motif enrichment analysis (MEA) was performed with Pscan[10]. Over-represented TFs were identified considering "mouse species" option, and with a Bonferroni p-value<0.05. *Zfp423* gene network was generated using "Gene Mania plugin" for Cytoscape [2] using co-expression option, and using as input file a list of genes whose change was detected upon *Zfp423* overexpression in the AstroEGFR*.
Proportion of tumor-retained gene expression in PT from AstroEGFR* specific cluster was computed by comparing the expression level of each gene in one sample against the mean of expression of the other samples.
Statistical analysis was performed using R statistical software (https://www.r-project.org/).
Principal component analysis was performed using FactoMineR package[11].

Supplementary References

1 Trapnell, C. *et al.* Differential gene and transcript expression analysis of RNA-seq experiments with TopHat and Cufflinks. *Nat Protoc* **7**, 562-578, doi:10.1038/nprot.2012.016 (2012).

2 Montojo, J. *et al.* GeneMANIA Cytoscape plugin: fast gene function predictions on the desktop. *Bioinformatics* **26**, 2927-2928, doi:10.1093/bioinformatics/btq562 (2010).

3 Langmead, B., Trapnell, C., Pop, M. & Salzberg, S. L. Ultrafast and memory-efficient alignment of short DNA sequences to the human genome. *Genome Biol* **10**, R25, doi:10.1186/gb-2009-10-3-r25 (2009).

4 Zhang, Y. *et al.* Model-based analysis of ChIP-Seq (MACS). *Genome Biol* **9**, R137, doi:10.1186/gb-2008-9-9-r137 (2008).

5 Thorvaldsdottir, H., Robinson, J. T. & Mesirov, J. P. Integrative Genomics Viewer (IGV): high-performance genomics data visualization and exploration. *Brief Bioinform* **14**, 178-192, doi:10.1093/bib/bbs017 (2013).

6 Heinz, S. *et al.* Simple combinations of lineage-determining transcription factors prime cis-regulatory elements required for macrophage and B cell identities. *Mol Cell* **38**, 576-589, doi:10.1016/j.molcel.2010.05.004 (2010).

7 Eisen, M. B., Spellman, P. T., Brown, P. O. & Botstein, D. Cluster analysis and display of genome-wide expression patterns. *Proc Natl Acad Sci U S A* **95**, 14863-14868 (1998).

8 Saldanha, A. J. Java Treeview--extensible visualization of microarray data. *Bioinformatics* **20**, 3246-3248, doi:10.1093/bioinformatics/bth349 (2004).

9 Subramanian, A. *et al.* Gene set enrichment analysis: a knowledge-based approach for interpreting genome-wide expression profiles. *Proc Natl Acad Sci U S A* **102**, 15545-15550, doi:10.1073/pnas.0506580102 (2005).

10 Zambelli, F., Pesole, G. & Pavesi, G. Pscan: finding over-represented transcription factor binding site motifs in sequences from co-regulated or co-expressed genes. *Nucleic Acids Res* **37**, W247-252, doi:10.1093/nar/gkp464 (2009).

11 Le, S., Josse, J. & Husson, F. FactoMineR: An R package for multivariate analysis. *J Stat Softw* **25**, 1-18 (2008).
